# Nanomaterials‐based sensors for the detection of COVID‐19: A review

**DOI:** 10.1002/btm2.10305

**Published:** 2022-04-13

**Authors:** Gowhar A. Naikoo, Fareeha Arshad, Israr U. Hassan, Tasbiha Awan, Hiba Salim, Mona Z. Pedram, Waqar Ahmed, Vaishwik Patel, Ajay S. Karakoti, Ajayan Vinu

**Affiliations:** ^1^ Department of Mathematics and Sciences College of Arts and Applied Sciences, Dhofar University Salalah Sultanate of Oman; ^2^ College of Engineering, Dhofar University Salalah Sultanate of Oman; ^3^ Faculty of Mechanical Engineering‐Energy Division K.N. Toosi University of Technology Tehran Iran; ^4^ School of Mathematics and Physics, College of Science University of Lincoln Lincoln UK; ^5^ Global Innovative Center for Advanced Nanomaterials College of Engineering, Science and Environment, The University of Newcastle Callaghan Australia

**Keywords:** biosensors, coronavirus sensor, COVID‐19, nanomaterial‐based biosensors, pandemic, point of care diagnosis, SARS‐CoV‐2

## Abstract

With the threat of increasing SARS‐CoV‐2 cases looming in front of us and no effective and safest vaccine available to curb this pandemic disease due to its sprouting variants, many countries have undergone a lockdown 2.0 or planning a lockdown 3.0. This has upstretched an unprecedented demand to develop rapid, sensitive, and highly selective diagnostic devices that can quickly detect coronavirus (COVID‐19). Traditional techniques like polymerase chain reaction have proven to be time‐inefficient, expensive, labor intensive, and impracticable in remote settings. This shifts the attention to alternative biosensing devices that can be successfully used to sense the COVID‐19 infection and curb the spread of coronavirus cases. Among these, nanomaterial‐based biosensors hold immense potential for rapid coronavirus detection because of their noninvasive and susceptible, as well as selective properties that have the potential to give real‐time results at an economical cost. These diagnostic devices can be used for mass COVID‐19 detection to understand the rapid progression of the infection and give better‐suited therapies. This review provides an overview of existing and potential nanomaterial‐based biosensors that can be used for rapid SARS‐CoV‐2 diagnostics. Novel biosensors employing different detection mechanisms are also highlighted in different sections of this review. Practical tools and techniques required to develop such biosensors to make them reliable and portable have also been discussed in the article. Finally, the review is concluded by presenting the current challenges and future perspectives of nanomaterial‐based biosensors in SARS‐CoV‐2 diagnostics.

## 1 INTRODUCTION

Coronavirus, COVID‐19, is an RNA virus that causes respiratory distress with pneumonic symptoms in humans and is first reported in Wuhan, a city in China, in December 2019. Since then, over 175 million people have been afflicted with this disease. Zhou et al. first discovered that an RNA virus is responsible for this infection. The sequencing of RNA extracted from the fluid of broncho‐alveoli of the diseased patients^1^ showed that this virus is closely related to severe acute respiratory syndrome (SARS).^2^ This virus infection results in a disease condition called the COVID‐19 infection. Now that we have reached one and half year mark of the pandemic, many researchers are still racing to find the most suitable and effective vaccine to treat the disease completely. However, challenges persist, owing to the growing number of mutations in the virus. The COVID‐19 virus remains inactive for 2–7 days, after which the infection further spreads within the body. During this inactive phase, the virus spreads rapidly from the infected patient to an uninfected individual and is considered the most contagious period.^3^ Due to the inability to analyze and quantify this viral infection rate, the degree of the pandemic remains uncertain.^4^ Apart from causing health distress, the virus has also caused significant havoc in the financial and social lives of millions of people around the globe.^5^ Therefore, the rapid diagnosis is highly critical to reduce the rate at which the virus is transmitting. The unprecedented time calls for more research to understand its epidemiology to create better‐targeted diagnostics and therapeutics.

While the conventional molecular diagnostics and microscopy‐based detection of virus infections have existed for a long time, sensing of analytes using electrochemical, colourimetric, or chemiluminescence methods are a great cost‐effective alternative for rapid detection of the SARS‐CoV‐2 with high sensitivity and selectivity.^6^ Several metal, nonmetal, and carbon‐based materials can be used to develop such biosensors.^7^ Among several materials available for their biosensing applications, nanomaterials are highly promising as they impart high selectivity and sensitivity to the sensor electrodes owing to their larger surface area that presents a large number of active sites for trapping or reacting with the analytes.^8^, ^9^ Nanomaterials like graphene and its derivatives,^10^ carbon nitrides,^11^ and gold^12^ can be effectively employed to develop biosensors for coronavirus detection. In addition, such sensors have the potential to be miniaturized and be more user friendly. Therefore, nanomaterial‐based sensors can serve as beneficial point of care devices and give reliable results even outside laboratory settings, thus also benefiting people in secluded areas.

The National Institutes of Health strategic plan for COVID‐19 research calls for the need “to improve the basic understanding of SARS‐CoV‐2 and COVID 19 and develop the necessary tools and approaches to diagnose, prevent, and treat this disease,” highlighting the impetus on the rapid and reliable diagnosis of the coronavirus.^13^ Thus, in the past couple of years, a lot of work has been conducted on rapidly detecting COVID‐19 using novel biosensors.^14^, ^15^, ^16^, ^17^, ^18^, ^19^, ^20^, ^21^ Likewise, many prominent researchers have spent a lot of time compiling several review articles about recent COVID diagnosis and treatment using different sensing strategies.^7^, ^22^, ^23^, ^24^, ^25^, ^26^, ^27^, ^28^, ^29^, ^30^ However, to the best of our knowledge, there is no systematic review article that extensively sums up the recent developments and research on nanomaterial‐based biosensors for rapid, sensitive, and selective detection of the SARS‐CoV‐2. Thus, the purpose of the current article is to provide a comprehensive overview of the recent advances in nanomaterials‐based biosensors for the detection of COVID‐19. First, a brief overview of the impact of coronavirus across the different countries in the world is discussed, followed by present testing methods for COVID‐19 and their shortcomings. We then discussed the recent studies on novel biosensors for SARS‐CoV‐2 detection and other nanomaterials‐based sensors that can be used for virus detection. A comparison of the nanomaterial‐based sensors to other conventional sensors are presented along with a brief section on other recent, relevant discoveries in this field of COVID diagnostics. Finally, the review is concluded with the present challenges and future perspectives of this emerging field.

### 1.1 Impact of COVID 19 across the world

On December 31, 2019, the first case of pneumonia with an undetermined reason was recognized in Wuhan city of China and was reported to the World Health Organization (WHO) office in China. This was tracked to the vendors and dealers of Huanan Seafood market of Hubei province in China and gained the attention of WHO officials who monitored the number of cases and prevailing conditions persistent because of the coronavirus situation. Since then, hundreds of scientific investigation and studies have been carried out in different parts of the world—each racing to find a permanent solution to end the COVID‐19 pandemic situation.

**FIGURE 1:**
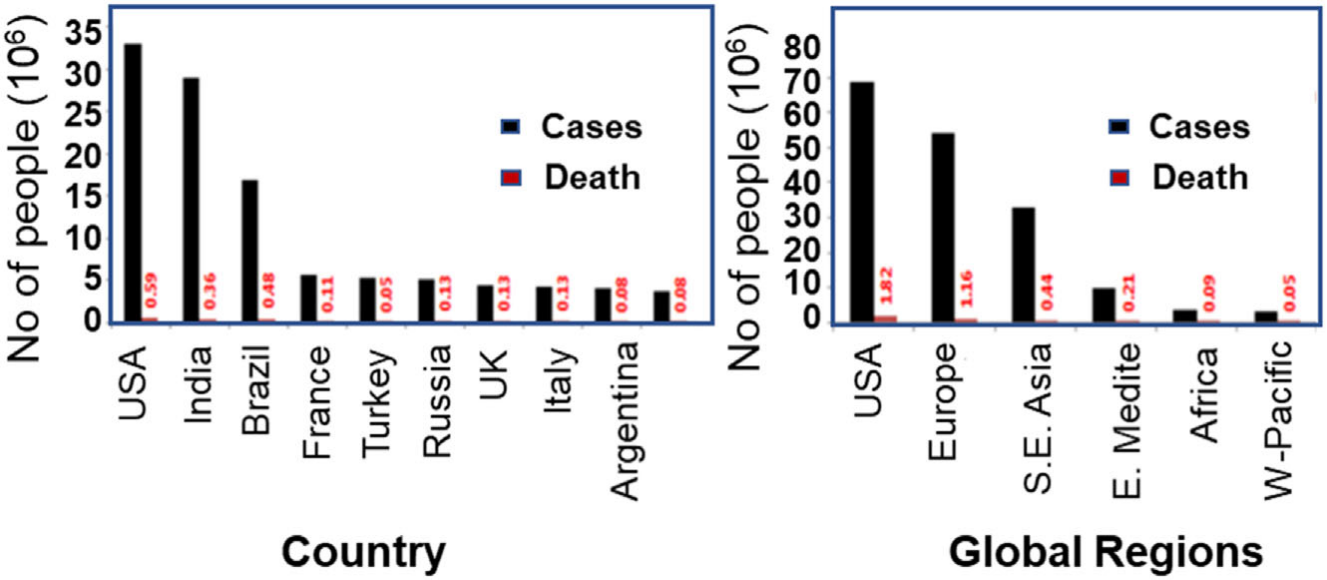
Trends in the number of cases and death due to coronavirus across different countries and global regions (data collected from WHO official website on June 11, 2021)

Since December 2019, among the 175.2 million afflicted individuals of COVID‐19 from 222 countries worldwide, the lives of more than 3.8 M people have been lost due to this COVID‐19 pandemic. The maximum confirmed COVID‐19 cases had been diagnosed in America (69,131,242), followed by Europe (54,828,356), and South‐East Asia (33,213,135). The Eastern Mediterranean, African, and Western Pacific countries have shown the minimum number of diagnosed coronavirus cases. Likewise, the maximum number of deaths was observed in the Americas (1,816,357) and then in Europe (1,162,992), followed by South East Asia (443,539). Among the countries, the maximum number of confirmed cases has been discovered in the United States (33,094,965), followed by India (29,274,823), Brazil (17,122,877), France (5,621,275), Turkey (5,306,690), Russia (5,167,949), United Kingdom (4,535,758), Italy (4,237,790), Argentina (4,038,528), Spain (3,715,454), Germany (3,709,129), and Colombia (3,635,835) (Figure 1).

### 1.2 Test methods for COVID‐19 detection and their challenges

**FIGURE 2:**
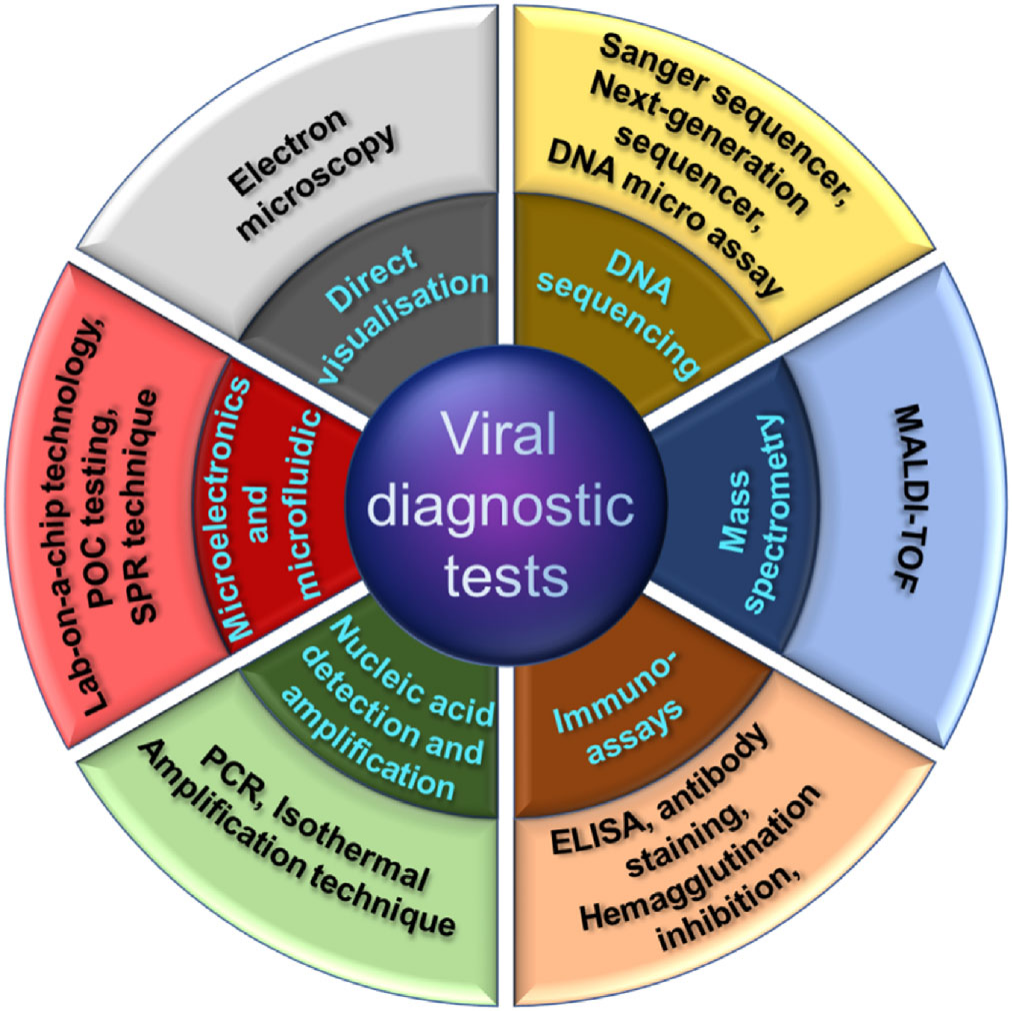
Diagram representing the options available for viral diagnostics

Considering the danger associated with the COVID‐19 pandemic and its contagious nature, the research on the detection and the cure for this dangerous disease has been gaining momentum. There are many existing options available for the detection of viral infection, as shown in Figure 2. Cell culture techniques are conventional methods that can detect viruses; however, this method does not have high specificity and involves several time‐consuming steps.^31^ On the other hand, the electron microscopy technique is an essential viral diagnostic tool and helps overcome any inconsistencies observed during the virus detection process. However, drawbacks like relatively lower detection sensitivity, extremely time consuming, and costly instrument dependence make this option problematic for the detection of COVID‐19. Likewise, due to similar drawbacks, methods like next‐generation gene sequencing and enzyme‐linked immunosorbent assay have not gained much popularity for virus detection.

Among the molecular testing options, polymerase chain reaction (PCR) micro devices and their testing methodologies have become widely popular in the past two decades. Of these devices, chamber PCR^32^, ^33^ and continuous flow PCR^34^, ^35^ have gained much attention from the scientific community. The recent half‐decade has witnessed the development of PCR chips based on silicon glass,^36^ SU‐8,^37^ polymethylmethacrylate (PMMA),^33^, ^38^ and polydimethylsiloxane (PDMS),^39^, ^40^ among others. These devices are rapid and possess the sample‐in answer‐out capability for COVID‐19 testing. However, there are inevitable disadvantages like the lack of accuracy, long incubation time, and considerable scope for errors.

**FIGURE 3:**
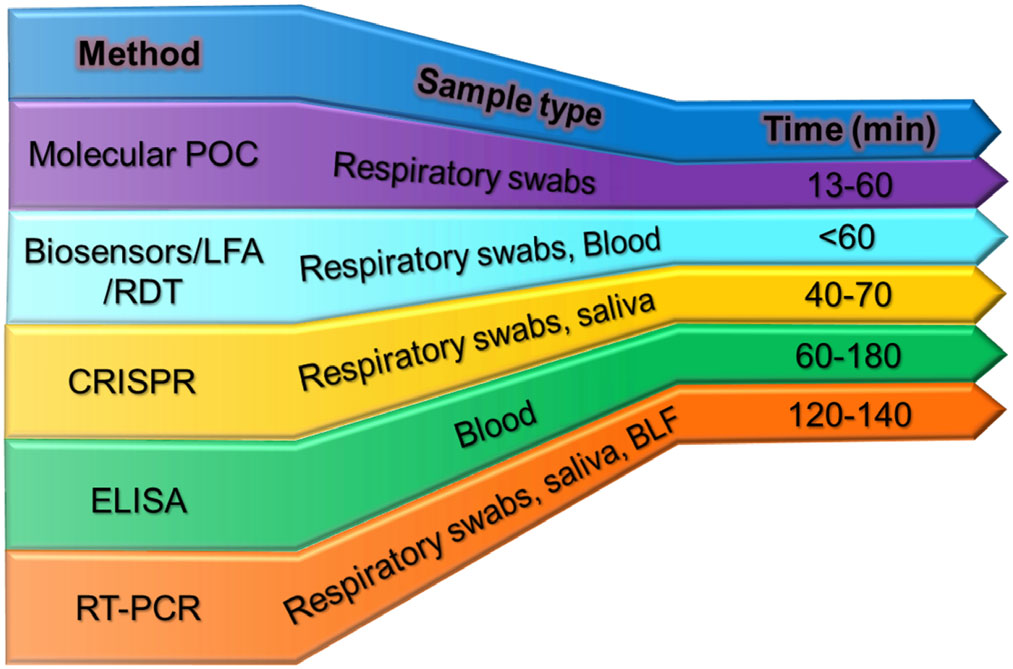
The comparison of major COVID‐19 diagnostic methods

One of the many challenges that this virus poses is the inability to identify an asymptomatic yet infected person and measure the amount of virus cast off from an infected body. Especially in a pandemic situation, it is crucial to understand the spread of infection for making better diagnostic tests available immediately, such as the point of care sensors with rapid detection capability. With the point of care devices, the treatment options for the patients can be improved, and effective therapies can be provided. In this sense, sensors have been an enormous benefit in increasing the testing phase rate^30^ and support the testing of a large number of patients. Furthermore, with thousands of global coronavirus cases detected daily, time‐efficient strategies for virus detection must be given more preference. Figure 3 shows a comparison of diagnosis time of various coronavirus testing methods that points to a clear need for biosensors with rapid diagnosis capability.

**FIGURE 4:**
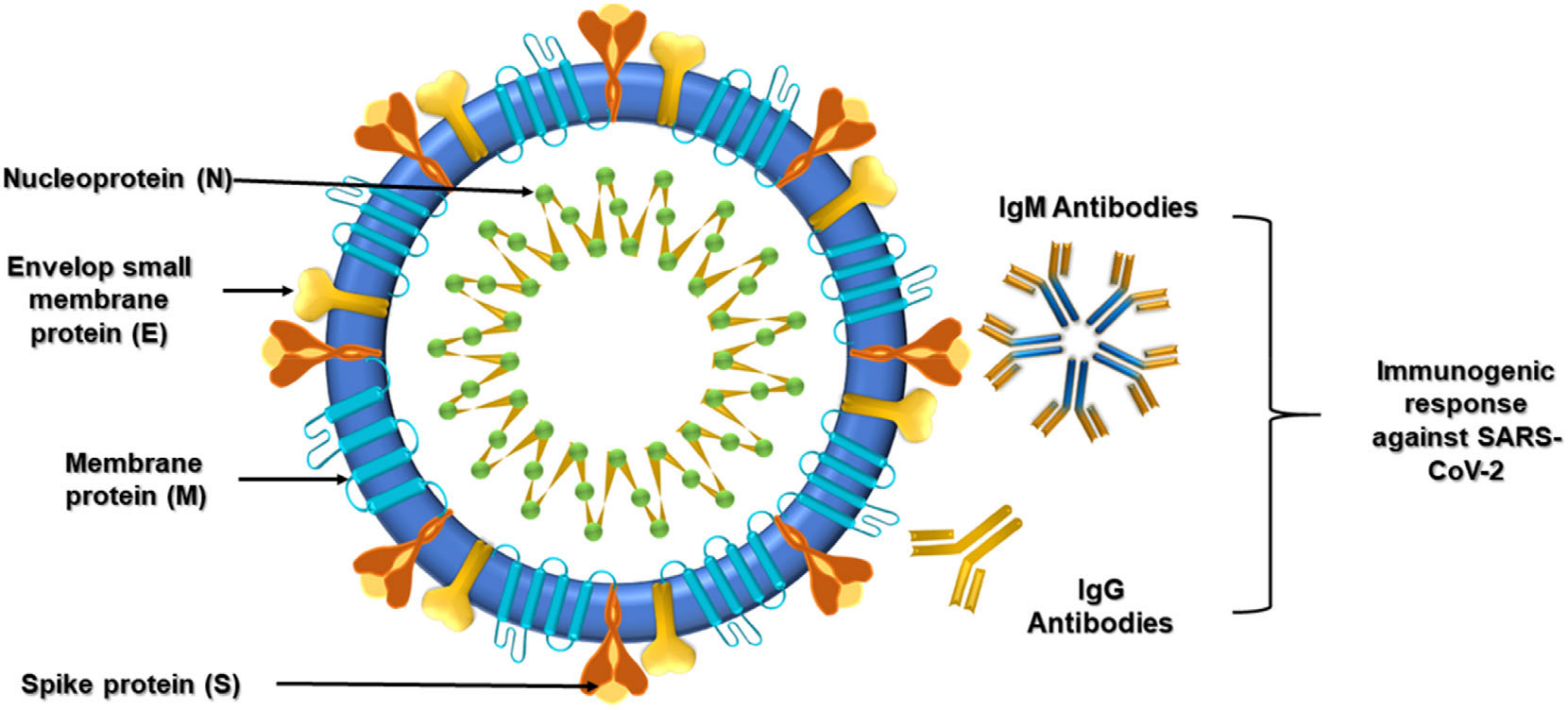
Schematic structure of SARS‐Cov‐2 and its possible target sites that can be used for biosensing and diagnosis

A biosensor consists of three major components: the receptor that interacts with the target analyte, a transducer that converts the signals into physically quantifiable output, and an electronic component that displays the output. The receptor ensures a strong binding with the analyte, giving it the specificity to identify and measure the target molecule. Depending upon the type of interaction with the analyte of interest, the transducer provides electrical, optical, or thermal signals that are recorded and displayed by the digital processor. The current bio‐sensing options allow the detection of biomolecules, including viral nucleic acids (DNA and RNA), viral proteins, and antibodies (Figure 4), produced by the infected individual's immune system to fight the virus.^41^ There are many available options of biosensors that can be used in the detection of viral‐based respiratory infections. Coupled with the remarkable physical, chemical, and mechanical properties of nanomaterials, biosensors shall become a fundamental tool in the field of viral theranostics.^42^, ^43^, ^44^

## 2 CHALLENGES WITH PCR‐BASED METHODS FOR THE DETECTION OF COVID‐19

Currently, the nucleic acid test is the principal methodology employed for coronavirus testing.^45^ RT‐PCR is popular in the testing kits currently available to diagnose coronavirus in suspected patients. The basic process behind SARS‐CoV‐2 testing through this kit is the reverse transcription of the virus RNA into its complementary DNA using specific enzymes. After this, particular regions of the complementary DNA are amplified to detect the virus's existence. However, the currently available PCR testing techniques require sophisticated laboratory and apparatus that are usually not available everywhere.^46^

Consequently, the transportation of the nasal swab or other COVID‐19‐related samples becomes necessary. Hence, in many instances, it may take up to 3 or more days to produce results, even though the actual testing of the sample may only take a few hours. Moreover, due to the number of steps involved in sample handling and transportation at various levels, there is a considerable risk of sample contamination or accidental spread of the virus. Additionally, the viral nucleic acid presence is not a direct indication of the severity of the disease,^47^, ^48^ an essential factor in the clinical diagnosis of a patient needing further medical attention.

Another added challenge to micro‐PCR testing is interfering molecules that sometimes give false‐positive or false‐negative results. Since over 30% of confirmed cases had been observed to be symptomless,^49^, ^50^ a high false‐negative rate will rapidly spread the disease within the community. Such false results are mainly attributed to unfavorable conditions of sample handling and transportation.^51^, ^52^ The samples usually collected from the patients are in the form of nasopharyngeal, anterior nasal, and midturbinate swabs.^53^ Recently, there have also been reports^54^ that suggest the possibility of iatrogenic CSF leakage due to nasal swab testing for COVID‐19. These limitations are the primary driving force toward finding other alternatives for nasal screening, especially in individuals with a history of skull base defects, surgery, or erosions, or even in patients with a history of sinuses. Studies suggest a risk of about 5%–10% of tested individuals who may have the case of epistaxis followed by a nasal swab test.^55^ Another study has reported an increased risk of epistaxis in nursing home residents. As many as 50% of people were affected, they were treated with oral anticoagulants.^56^ This triggers a significant attention toward less invasive testing methods like saliva sampling or midturbinate testing.

To reduce the severity of nasal swab testing for SARS‐CoV‐2 detection, saliva‐based tests have also been encouraged.^57^ The saliva collection process is noninvasive and does not produce aerosols. Moreover, the testing does not require professionals, and the testing can be done in simple and easy steps, even in remote areas. However, studies also indicate that there may be lower detection rates of COVID‐19 from saliva samples (63%) when compared to the samples of bronchoalveolar lavage fluid (93%).^58^ Hence, saliva‐based testing for SARS‐CoV‐2 diagnosis will need further testing to confirm the patient's infection status.

As discussed above, the currently popular RT‐PCR and related techniques provide delayed results and disallow on‐site diagnosis. Other shortcomings include their efficiency to diagnose cases in the early stages, cumbersome sample preparation and purification process that are time‐consuming.^59^ Most of the procedures require many complex apparatuses, skilled laboratory technicians, and additional requirements that increase the overall expense of these methods. Hence, better and more efficient methodologies are quickly needed to detect and analyze the virus and its antibodies, taking the diversity and the viral replication niches into account. Also, these diagnosis methods must be user‐friendly for early detection of the viral infection before the first signs of the symptoms set in to reduce the spread of the viral infection. These issues can be overcome by biosensors‐based detection as they can be far more effective than traditional methods due to their ability to give rapid results with high sensitivity, selectivity, and specificity even in very low sample concentration.^60^

## 3 NOVEL BIOSENSORS FOR THE DETECTION OF COVID‐19

**FIGURE 5:**
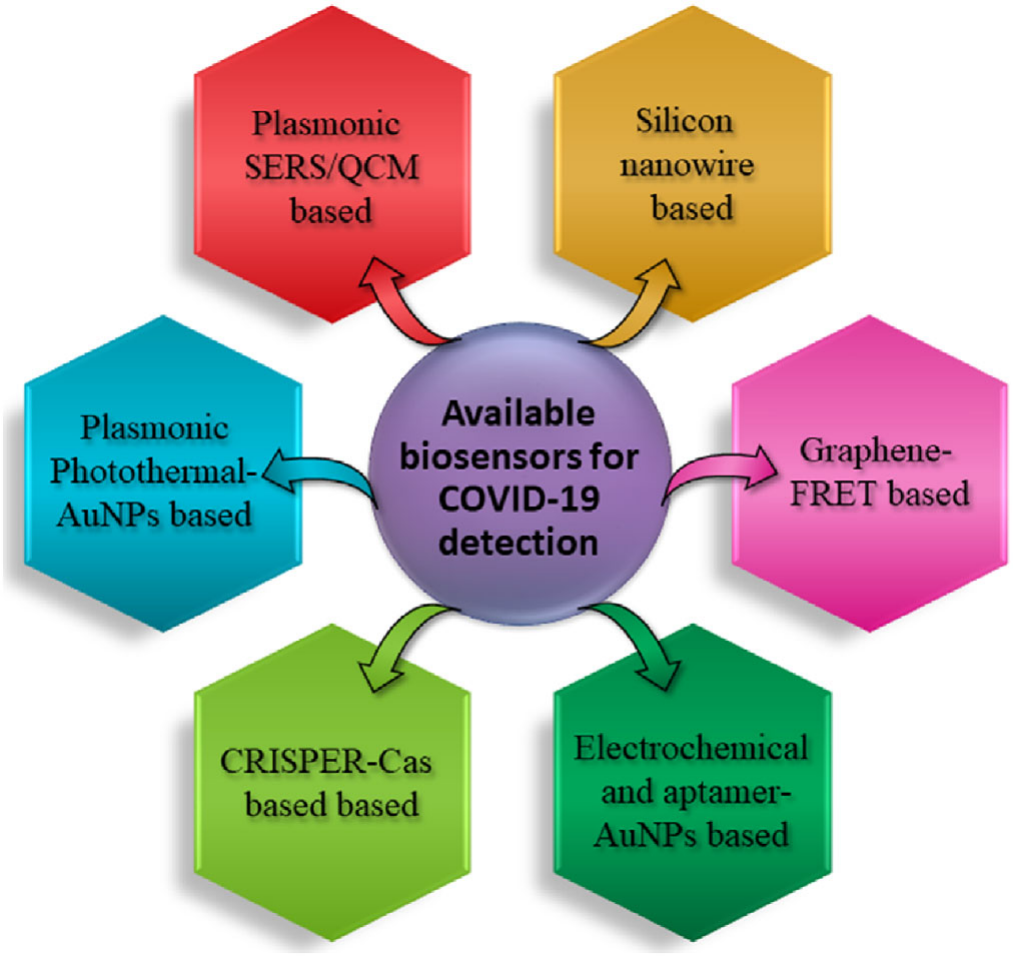
Biosensors used for SARS‐CoV‐2 detection and diagnosis

Sensors can play a fundamental role in reducing the time taken to get coronavirus testing results, especially during this pandemic. Biosensors are devices that are integrated with the transducer and detector recognize biomolecules like enzymes, nucleic acids, and antibodies. Different types of biosensors are currently being researched to detect the COVID‐19 infection and are listed in Figure 5.

**FIGURE 6:**
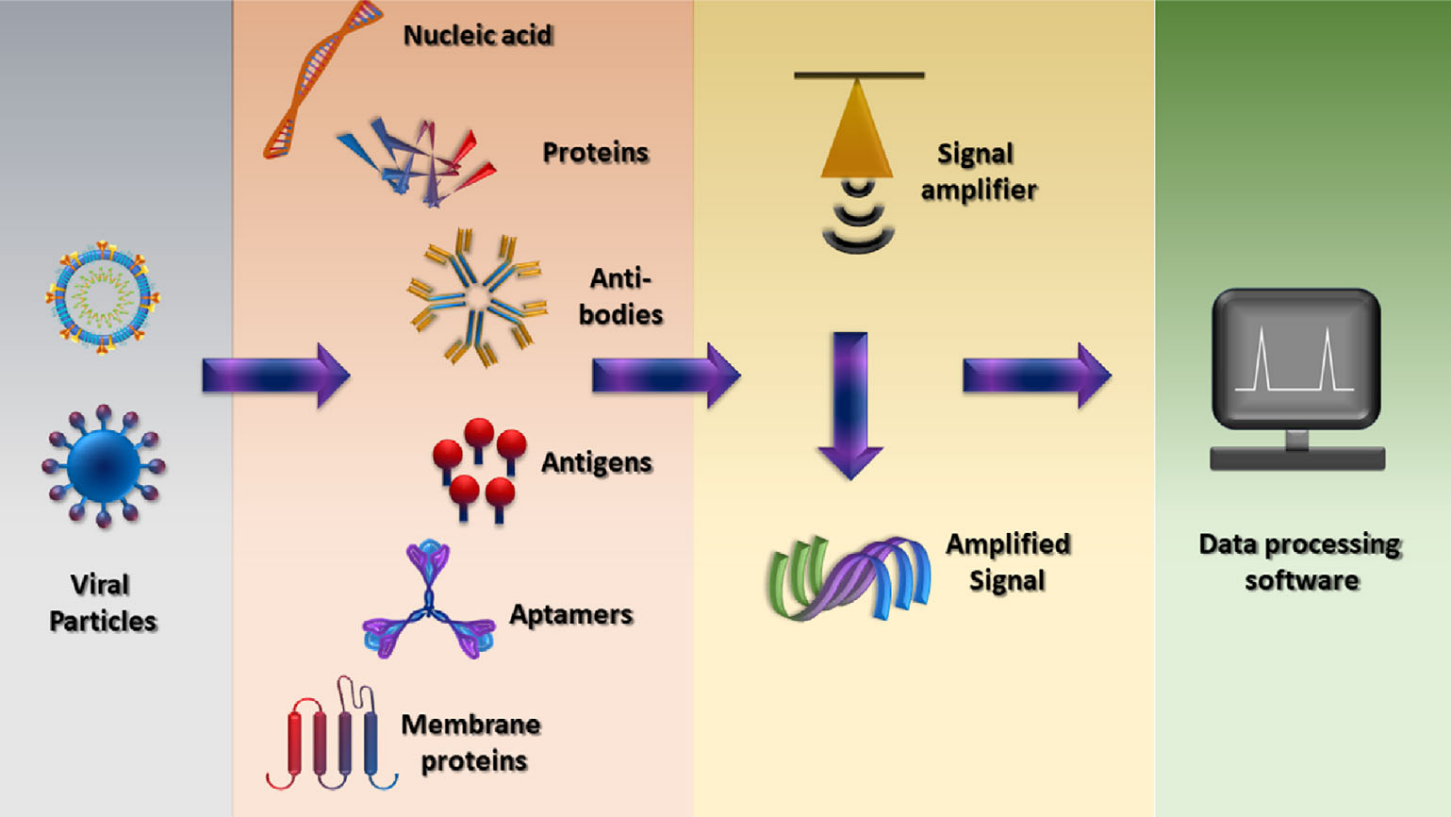
Schematic diagram representing the parts of a biosensor for COVID‐19 (analyte) detection

Viral biosensors recognize virus targets and can be categorized into five types based on their ability to identify the recognition element. These include immunosensor, DNA‐based biosensor, antigen‐based biosensor, cell‐based biosensor, and molecular imprinting‐derived biosensors.^31^, ^60^, ^61^, ^62^ These biosensors are promising entities against conventional therapeutics. Viral biosensors have specific advantages over conventional molecular diagnosis because of their affordability, sensitivity, ability to generate quick results, small size, and ease in carrying testing anywhere.^63^ Due to the recent progress in genetic engineering, transduction systems, and nanobiotechnology, viral biosensors have seen an intense surge in their diagnostic and therapeutic applications.^44^, ^64^ Depending on the kind of biosensing application, biosensors for respiratory illness can be divided into four distinct types: optical, electrochemical, piezoelectric, and thermal biosensors^65^, ^66^ (Table 1). On several occasions, researchers have combined traditional molecular‐based methods with nanotechnology or added an unconventional quantification technique to the molecular methods for increasing the efficiency and speed of detection. Monoclonal antibodies, nucleic acids, proteins, antibodies, and aptamers can all be employed for the specific recognition of SARS‐CoV‐2 infection (Figure 6). A further description of different types of sensors in each category is provided in the following sections.

**TABLE 1:** Different types of available biosensors for virus detection and diagnosis

No.	Types of biosensor	Types of cirus	Specific recognition site	Detection of other viruses
1.	Immuno‐electrochemical	Influenza A	M1 protein	Parainfluenza, Rhinovirus, MERS, SARS‐CoV
2.	Immuno‐optical	MERS	Recombinant spike protein S1	SARS‐CoV, H5N1 influenza virus, Human adenovirus, Respiratory syncytial virus
3.	Thermal	SARS‐CoV	RNA dependent RNA polymerase	MERS, SARS‐CoV‐2
4.	Piezoelectric	SARS‐CoV	Spike protein	Influenza virus, Adenovirus, RSAV, MERS

### 3.1 Loop‐mediated isothermal amplification‐based sensors

The viral genomic RNA, spike glycoproteins, and membrane proteins, when bound to the host ACE‐2 receptors, elicit rapid humoral immune response^67^ mediated by IgM and IgG antibodies. These antibodies can be used to identify the foreign COVID‐19 infection prevailing inside the body.^68^ The drawbacks posed by the conventional qRT‐PCR‐based assays are combated using the reverse transcription loop‐mediated isothermal amplification (RT‐LAMP) method.^69^, ^70^, ^71^ This was demonstrated in work put forward by Zhu et al. who studied the single‐step RT‐LAMP‐assisted nanoparticles‐based biosensors (NBS), creating the RT‐LAMP‐NBS assay for the specific and quick detection of COVID‐19^72^ using the F1ab LAMP primer sets. The nucleoproteins of coronavirus were multiplied and analyzed in a single step through the NBS. Their work demonstrated that the RT‐LAMP‐NBS detected SARS CoV‐2 with high specificity and selectivity. The assay's sensitivity was observed to be 12 copies per reaction, which made the amplification process far more efficient and reduced the chances of errors during diagnosis. The authors also concluded that the assay had 100% sensitivity against coronavirus detection in clinical samples and took less than an hour for the results.^72^ Thus, this sensor has the translational potential for detecting and monitoring coronavirus in clinical settings.

In another study by Amaral et al., developed a single tube test based on RT‐LAMP that allowed visual detection of less than 100 SARS‐CoV‐2 genome copies within 30 min.^73^ A comparison of the tube test against RT‐PCR test, commonly used for COVID‐19 detection, using 177 nasopharyngeal RNA samples showed that the tube test displayed 100% sensitivity with a specificity of 96.1%. The same researchers also developed an RNA extraction‐free RT‐LAMP test that allowed for virus detection from saliva by creating an alternate pathway that used DNA extraction columns to reduce the expenses associated with RNA extraction.

LAMP reaction thus provides a time‐efficient pathway that successfully overcomes the intricate and laborious processes in traditional detection techniques like PCR (Table 2). Under isothermal conditions, this technique rapidly multiplies nucleic acid with high efficiency and specificity and is more favorable for creating point‐of‐care devices for COVID‐19 detection.^74^ Thus, LAMP‐based sensors can be potentially applied for clinical applications toward the SARS‐CoV‐2 detection and monitoring.

**TABLE 2:** Comparison between PCR and LAMP techniques

Parameters	PCR	LAMP
Required time	Need specific sample concentration and preparation step. Hence, time‐consuming.	Simplified sample preparation. Time‐efficient.
Protocol	Complicated protocol which requires a skilled technician	Single protocol which gives rapid results
Efficiency	Inhibitor hinders the reaction	Tolerate inhibitors and more stable
Sensitivity	Diagnostic sensitivity <95%	Diagnostic sensitivity >95%

### 3.2 Clustered regularly interspaced short palindromic repeats associated proteins‐based biosensors

The relatively new CRISPR system can also be used for SAR‐CoV‐2 detection and diagnosis.^75^ This method effectively spots bacteria, cancer mutations, and microRNAs by substituting target‐specific crRNA/sgRNA (the cas unit). Nanomaterials, because of their versatile properties, have been used alongside the system to create CRISPR‐based biosensors that can detect respiratory viruses. This was observed in the work of Hajian et al.,^76^ who developed a CRISPR‐chip biosensor associated with the graphene‐based field‐effect transistor (FET) to digitally detect a given target sequence present inside a genome. This CRISPR chip made the use of deactivated CRISPR‐Cas 9 complex linked to a specific single‐guide RNA and was attached to a transistor resulting in a label‐free nucleic acid sensing device whose signals could be analyzed using a simple reader. This highly sensitive biosensor could detect 1.7 fM of target nucleic acid within 15 min and without any external amplification and boasts to spot the covid infection in under 40 min. Based on the same principle, Broughton et al. created a lateral flow assay based on the CRISPR‐Cas‐12 gene to selectively detect the coronavirus protein in less than 40 min from clinical samples.^77^ The protein concentrations analyzed using this bioassay were further cross‐checked using respiratory RNA extract swabs of several dozen virus‐infected patients. This particular assay was reported to show high sensitivity at shallow levels (10 copies/μl input) and could exhibit a high selectivity of 95%–100% for noninfected individuals. Called the DETECTR assay, this method also works similar to the qRT‐PCR technique and has shown to have high accuracy like the PCR technique but at the same time has disadvantages synonymous with the qRT‐PCR such as availability of chemicals, reagents, and isolation and extraction kits. Thus, the CRISPR‐based assay using Cas‐12 genes allows high selectivity and can be used to successfully detect SARS‐CoV‐2 particles in human clinical samples.

### 3.3 Surface plasmon resonance‐based biosensors

SPR and localized SPR (LSPR)‐based biological sensors have also shown application in the detection of viral diseases^41^, ^78^ by detecting the viral nucleic acid particles and nucleocapsid antibodies against coronavirus in undiluted human serum. The thermoplasmonic SPR sensor is enveloped with a peptide monolayer, and the surface of the probe is functionalized with a recombinant protein of nucleocapsid present in SARS‐CoV‐2. The nucleocapsid protein is found in nM concentration within the anti‐SARS‐CoV‐2 antibodies. Djaileb et al., used a thermoplasmonic SPR sensor to design an assay that was quick and can be used as a label‐free assay for the detection of coronavirus in the sample under 15 min,^79^ therefore proving to be an effective sensing methodology for coronavirus testing.^80^ In another interesting study, Ahmadivand et al. developed a terahertz plasmonic meta sensor that can detect the SARS‐CoV‐2 spike proteins in femtomolar concentrations.^81^ The miniature sensor fabricated in this study was based on the toroidal electrodynamics concept, allowing them to develop the plasmonic modes in terahertz frequencies. Gold NPs functionalized with specific antibodies targeting the spike proteins on the surface of the virus was used as the active sensor material. The sensor depicted a LOD of ~4.2 fM and thus demonstrates a significant promise in translation of such sensors toward precise and rapid detection of the COVID virus. In another unique yet promising study by Sharma et al., a graphene oxide‐based double interdigitated capacitive biosensor was developed to detect the COVID spike proteins with high sensitivity.^14^ This GO/EDC‐NHS/anti‐SARS‐CoV‐2Abs‐based electroactive immunosensor demonstrated a high sensitivity of 1 fg/ml, a wide linear range of 1 mg/ml to 1 fg/ml, and ~3 s of response time. This portable device was functional up to 10 days when stored at 5°C and holds significant promise toward early diagnosis of the COVID virus in clinical samples.

### 3.4 Resistance‐based sensor array

**FIGURE 7:**
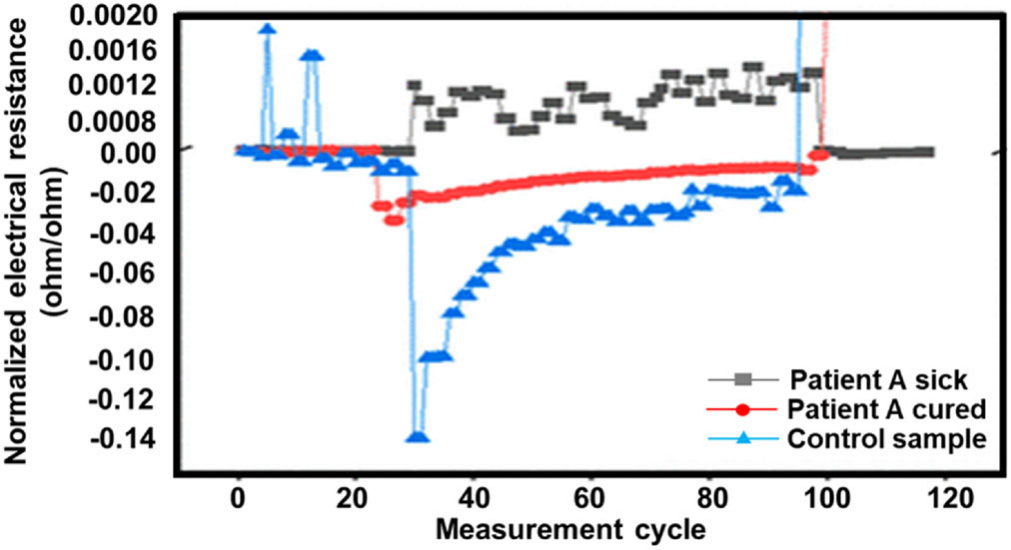
Sensor response of three different breath samples^12^

Shan et al. reported a nanomaterial‐based sensor array showing multiplexed properties for detecting and monitoring COVID‐19 from exhaled breath.^12^ This sensor comprises of a sensing layer that could expand or could contract in the presence of volatile organic compounds (VOCs). This, in turn, causes a change in the electrical resistance, which can be recorded. In this sensor, the inorganic nanomaterials facilitate the electrical conductivity, while the organic part of the film allows the adsorption of VOCs.^42^, ^82^, ^83^ When the analyte is exposed to this biosensor, the VOCs encounter the sensing layer and interact with the organic element, the functional group present in the inorganic nanomaterials. These interactions result in a change in the volume of the nanomaterial layer and create a difference in conductivity. The authors developed an array of eight gold nanoparticles based on the previously mentioned principle and integrated them within an electronic circuit. Their apparatus could collect a sample of exhaled breath when blown into the device for a couple of seconds from a distance of 1–2 cm. The authors noted that when the breath moved past the array, VOCs related to SARS‐CoV‐2 reacted with the sensors and released a set of electrical resistance signals as a function of time (Figure 7).

Several literature reviews reveal many such examples of sensors that imitate the functioning of natural mammalian olfactory systems and hence are of superior value in COVID‐19 detection and diagnosis.^82^, ^83^, ^84^, ^85^ Often these sensors play such a significant role because of their chemical diversity in terms of the functional groups present on the nanoparticles and hold an array of cross‐reactive semi‐selective sensory units. Another essential advantage of such arrays is their flexibility and unique role in identifying “fingerprints” of similar chemical patterns under varying conditions.^85^ The significant advantages proposed by these sensors show their promise toward detection of coronaviruses in clinical samples.

### 3.5 Point‐of‐care lateral flow immunoassay

Serological tests like LFIA have been fabricated for anti‐SARS‐CoV‐2 antibodies detection during COVID‐19 diagnosis.^86^, ^87^ These assays have several advantages, including low technical requirements, reduced time of diagnosis, inexpensive, and increased sensitivity and selectivity. This was observed in a rapid bioassay developed by Chen et al. to detect anti‐SARS‐CoV‐2 IgG for COVID‐19 diagnosis in human serum.^88^ The assay was based on a lanthanide‐doped polystyrene nanosystem, with detection time within 10 min. The authors used a nitrocellulose membrane to capture IgG via recombinant nucleocapsid phosphoprotein of the virus. A nanosystem tagged with IgG antibody of mouse plays the role of a fluorescent readout. This sensing platform has shown promising results and can be used for COVID‐19 disease management owing to its high selectivity, mobility, and inexpensiveness.

Lie et al. prepared AuNPs that could simultaneously spot IgM and IgG antibodies of COVID‐19.^89^ The authors developed a testing strip by using an NC membrane on which anti‐human‐IgM, anti‐human‐IgG, and anti‐rabbit‐IgG (control) were placed along three separate test lines. Next, a conjugation pad was brought in contact with a mixture containing AuNPs‐SARS‐CoV‐2 recombinant antigen conjugate and AuNPs rabbit IgG. This sensor could diagnose infection in under 15 min in any given human blood sample. The authors also reported that this LFIA sensor could also analyze different stages of SARS‐CoV‐2 disease in different patients. The sensor was shown to have a sensitivity of 88.66% and a high specificity of 90.63%.

Lanthanide possesses unique optical properties like sharp emission bands, which allows its use in sensing applications.^90^ For instance, Banerjee and Jaiswal reported an LFIA‐based biosensor to detect infectious agents.^91^ They used lanthanide‐doped NPs (LNPs) to develop the sensor. Chen et al. also developed a similar biosensor by mini‐emulsion polymerization to detect COVID‐19 particles in human serum samples.^88^ LNPs were functionalized via mouse antihuman IgG and rabbit IgI after EDC/NHS chemical reactions. The recombinant nucleocapsid phosphoprotein of COVID‐19 was attached to a nitrocellulose membrane on the sensor, resulting in the confinement of specific IgG. This LFIA method showed the potential to detect anti‐COVID‐19 IgG in under 10 min in a given human serum sample. The results of these studies conclude that they meet the necessary requirements for the clinical diagnosis of the virus, including rapid and accurate detection in less time; and thus can be used to monitor the SARS‐CoV‐2 progression and their response to different treatment options used.

### 3.6 FET‐based biosensor

In another study, Seo et al. used the graphene‐based nanomaterial and showed the effectiveness of FET‐based biosensors in coronavirus detection. The FET biosensors use graphene coating coupled with a monoclonal antibody,^10^ which can spot the SARS‐CoV‐2 spike proteins in nasopharyngeal swab with high sensitivity. The device could detect up to 1 fg/ml of SARS‐CoV‐2 spike protein in phosphate‐buffered solution and up to 100 fg/ml in clinical transport mediums. Additionally, this sensor showed a detection limit of 1.6 × 10^1^ pfu/ml for the virus proteins. Additionally, this sensor showed a detection limit of 2.42 × 10^2^ copies/ml for the virus proteins in clinical samples. This device could detect the SARS‐CoV‐2 with high sensitivity without any prior treatment or labeling and thus holds promising potential toward the development of POC devices toward COVID‐19 testing.

### 3.7 Dual functionalized gene‐based biosensor

Qui et al. designed and developed a dual function plasmonic SARS‐CoV‐2 gene‐based biosensor.^92^ The sensor involved the combined features of the plasmonic photothermal (PPT) effect in addition to LSPR‐based transduction. This unique biosensing approach gave enhanced sensitivity, which is highly favorable for COVID‐19 diagnostics. This research also made the use of a 2D nanostructure of gold nanoislands (AuNIs) as the plasmonic platform that was modified using complementary DNA to identify a fixed SARS‐CoV‐2 sequence employing gene hybridization. The AuNIs were altered after the self‐assembly of thermally de‐wetted gold nanofilm on a BK7 glass surface. The gold nanofilm was prepared by magnetron sputtering. The authors noted thermoplastic heat generation when gold was illuminated at a specific frequency during the sensing process. The sensing properties were enhanced by allowing the laser beam to fall at two different angles, which caused the plasmonic resonances of PPT and LSPR to excite at two unique wavelengths.^80^ This is because it entails in situ hybridization that allows for the specific identification of gene sequences. The in situ PPT enhancement results revealed that the hybridization kinetics was significantly improved and allowed for specific detection of nucleic acids, and could distinctly discriminate between the separate gene sequences. The authors further elaborated that this sensor showed a low detection limit of 0.22 pM and showed a high selectivity in SARS‐CoV‐2 detection, even in heterogeneous mixtures involving multiple genes. Also, the authors tested different receptor binding domains using monoclonal antibodies specific to SARS‐CoV and concluded that the Abs did not have a solid attachment to the spike proteins of the virus suggesting the superiority of this LSPR technique for clinical COVID diagnosis and monitoring.

### 3.8 Electrochemical sensors

**FIGURE 8:**
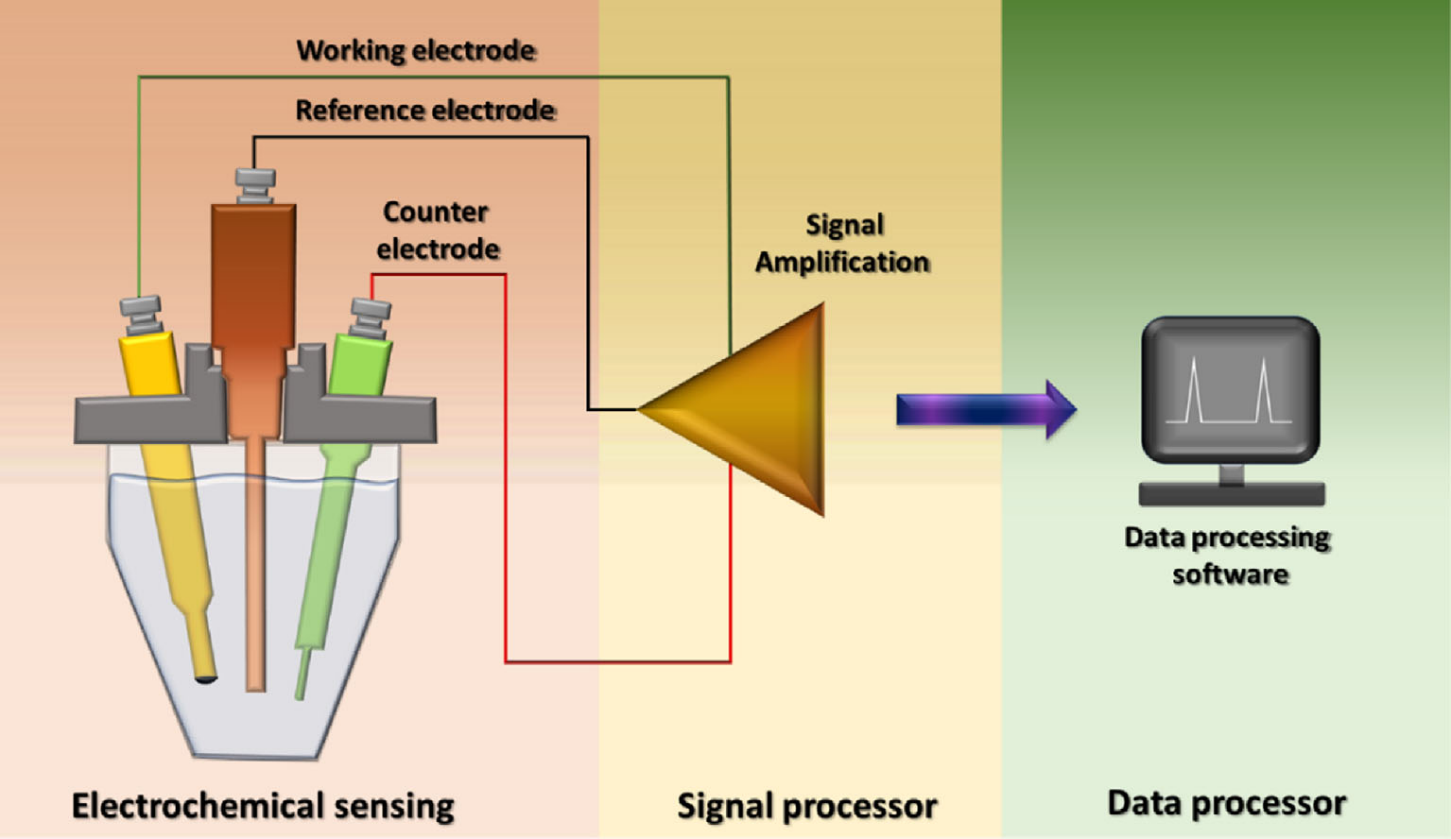
Schematic diagram representing the electrochemical sensor in a three‐electrode configuration

Electrochemical sensors have proven to be a highly suitable diagnostic tool for SARS‐CoV‐2 detection.^44^ These devices can analyze target molecules at picomolar concentrations.^93^ Electrochemical sensors can be used in many configurations for the detection of a molecule, such as a three‐electrode configuration depicted in Figure 8. At present, disposable immune‐sensing chips can be used in electrochemical biosensors to cut down the total cost of biosensors. Nanoparticles modified substrate and interdigitated electrodes can enhance the efficiency of the biosensors for coronavirus detection.^44^ These functionalized biosensors also amplify signals to allow room for a low detection limit and enable a more comprehensive biosensing range. Such a biosensing chip can be linked to a small potentiostat interfaced to a smartphone to give rapid, on‐site coronavirus results.

Mahari et al. employed a nanoparticle‐based electrochemical sensor to create an in‐house built sensor device, the eCovSens.^94^ The potentiostat‐based sensor uses a fluorine‐doped tin oxide (FTO) electrode attached to gold nanoparticles and nCOVID‐19 antibodies that could detect any changes in the electrical conductivity upon interaction with the virus antigens. In addition, a second set of carbon electrodes were developed by screen printing followed by the attachment of nCOVID‐19 antibodies to measure the changes in the conductivity. The activity of both the sensors was analyzed upon the interaction of nCOVID‐19 Ab with nCOVID‐19 Ags. Both immunosensors were observed to be very specific in detecting the nCOVID‐19 spike antigen and could detect the antigen within 1 fM to 1 μM concentrations. The device could quantify the results in saliva samples within 10–30 s with a LOD of 90 fM using the eCovSens and 120 fM using the potentiostat sensor.

Gold nanosystems have exceptional physicochemical properties that enable them to play a wide variety of roles, primarily as signal transducers in biosensors.^95^ Mahari et al. reported an electrochemical biosensor to detect spike S1 protein antigen of COVID‐19.^94^ The sensor was based on a fluorine‐doped tin oxide substrate containing a 29 nm AuNPs drop casted film as the signal amplifier. Monoclonal antibodies of SARS‐CoV‐2 were attached to the drop cast to give rise to an FTO/AuNPs/COVID‐19Ab immunosensor. Due to the interaction between COVID‐19 Ab and COVID‐19 Ag, the authors observed a change in the electrical conductivity that resulted in the enhancement of current measured for different concentrations (1 fM to 1 μM) COVID‐19 Ag. The detection limit of the sensor was observed to be 10 fM and showed excellent selectivity toward COVID‐19 Ag. The sensor was noted to be highly stable for up to 3 weeks.

### 3.9 Colorimetric biosensors

**FIGURE 9:**
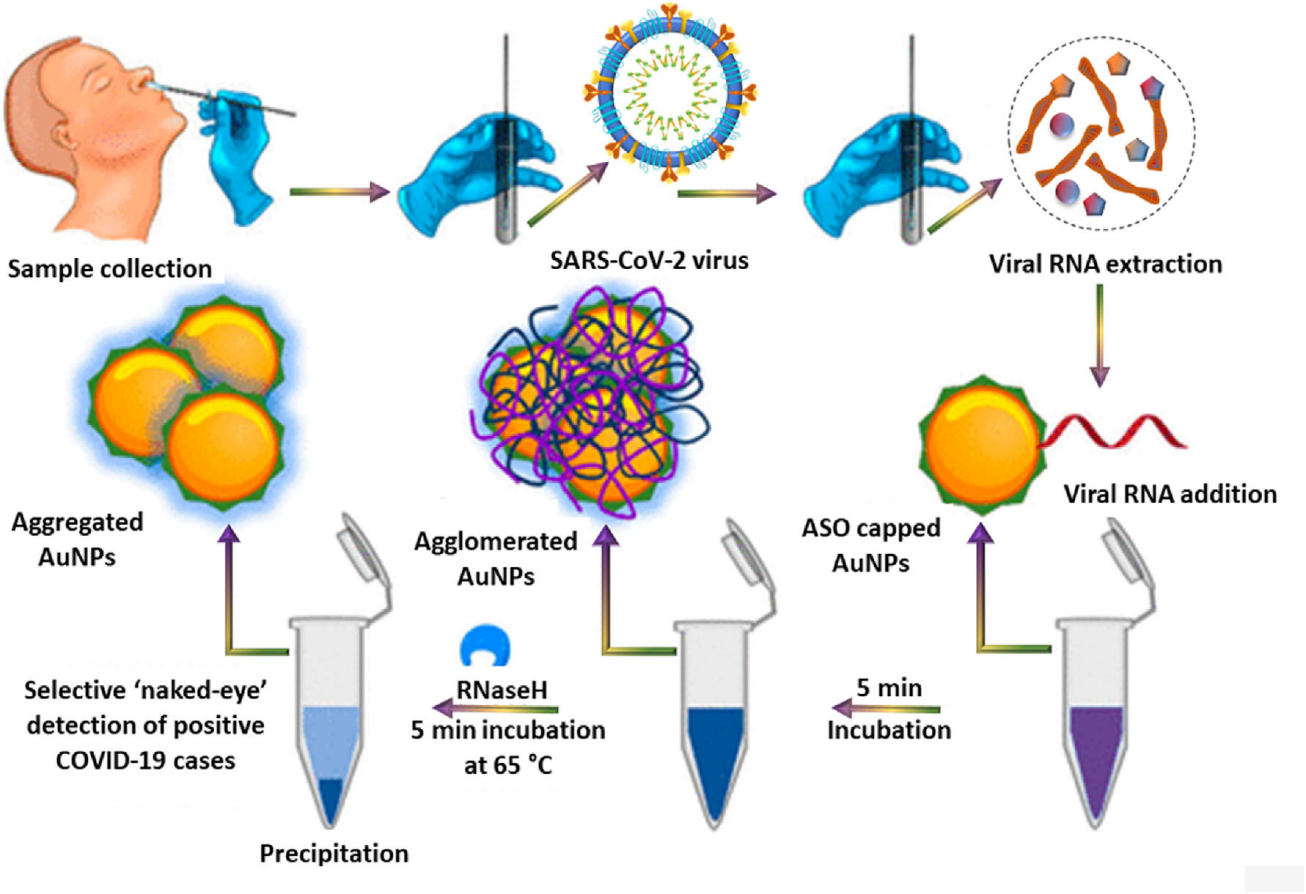
The selective naked‐eye detection of SARS‐CoV‐2 using modified gold nanoparticles^72^

Colorimetric assays are also used for the diagnosis of infectious viral diseases. They are reliable and straightforward because of the ability to detect the infection through the naked eyes. Moitra et al. prepared AuNPs linked to thiol modified antisense oligonucleotides (ASOs) to identify and detect SARS‐CoV‐2.^96^ Through this approach, the authors observed that the sensing was limited only to identify the nucleocapsid phosphor protein (N‐gene) from RNA sample present in an oropharyngeal swab, resulting in giving early results in under 10 min. The principle behind the colorimetric detection was the agglomeration of AuNPS‐ASOs nanostructures in the presence of target coronavirus RNA sequences, which caused a redshift in the UV‐absorbance due to the SPR effect. The authors also recorded that the strands in RNA–DNA hybrid separated upon the addition of RNaseH into the solution and resulted in their precipitation from the mixture, which was visible by naked eyes (Figure 9). The detection limit was noted to be 0.18 ng/μl for SARS‐CoV‐2 RNA, and the dynamic range was recorded as 0.2–3 ng/μl. Compared to all previously discussed studies, colorimetric assays are an excellent alternative for quick detection of the virus because of the ability to notice the changes in clinical samples even with naked eyes.

### 3.10 Magnetic nanoparticles‐based detection

Many types of MNPs have been employed to create biosensors for the diagnosis of different respiratory viruses.^97^, ^98^ These include techniques like magnetic resonance,^99^ fluorescence,^100^ rolling circle^101^ and electrochemical amplification.^102^ For example, Zhao et al. developed carboxyl polymer‐coated magnetic NPs (pcMNPs) and used this setup to extract viral RNA for accurate SARS‐CoV‐2 diagnosis.^103^ This system showed simultaneous properties like the lysis of viral particles and the binding of RNA in one step. Hence, the resultant pcMNPs‐RNA complexes are further employed in RT‐PCR reactions. The authors reported that this sensor could identify the ORFlab and R gene of the virus and perform RT‐PCR‐linked sensing. The sensor was also noted to be 10‐copy sensitive and showed linearity with the pseudovirus particles. Hence, the pcMNPs‐based RNA extraction approach is a better option for quick diagnosis of SARS‐CoV‐2 in clinical samples than the RT‐PCR‐based method.

## 4 OTHER NANOPARTICLE‐BASED SENSORS THAT CAN POTENTIALLY BE USED FOR COVID‐19 DETECTION

**FIGURE 10:**
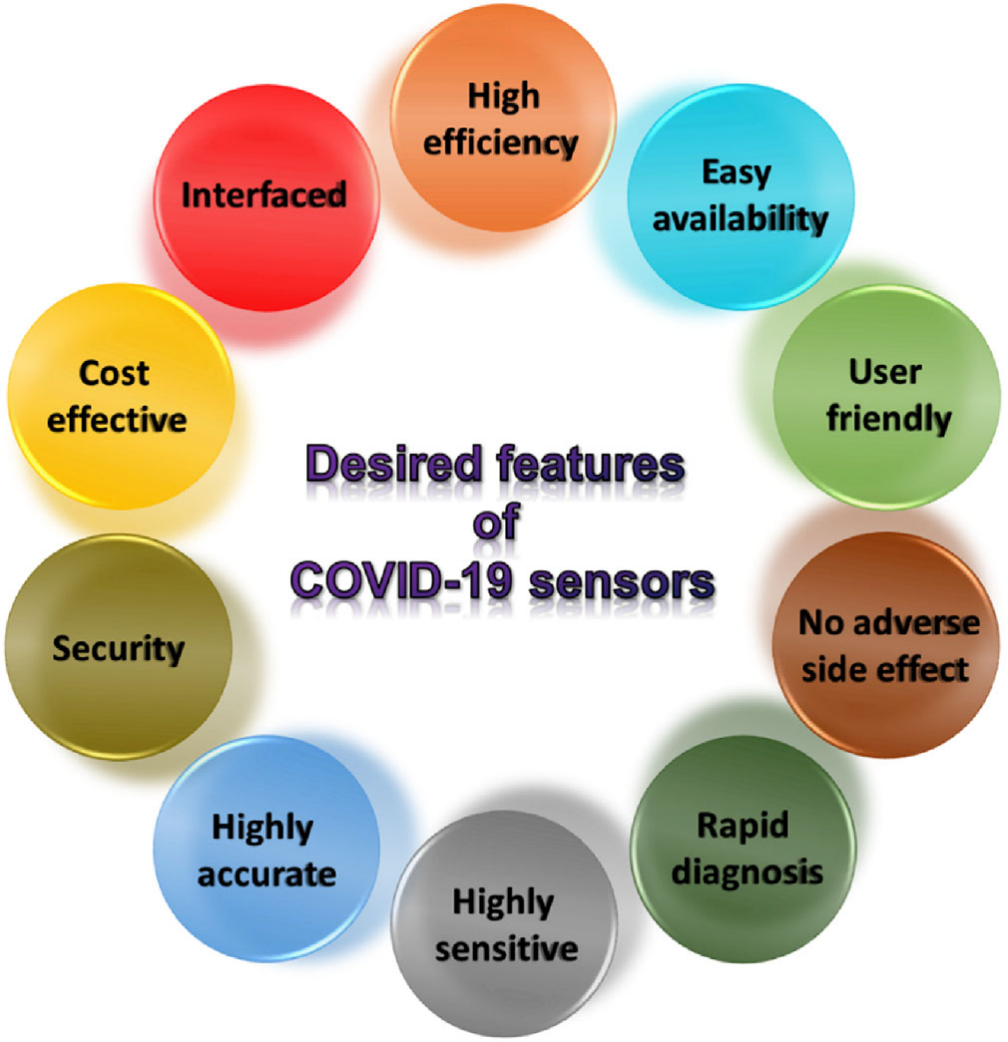
Factors to consider for biosensor development for early‐stage COVID‐19 diagnostics

The introduction of miniaturization and nanotechnology has enabled biosensors to achieve a fine structure and exhibit more sensitivity and selectivity against the target antigens/proteins, even when they are present in minute quantities. These possibilities allowed to meet many target features of a biosensor that are equally important in detecting Covid‐19 infection. Several factors have to be considered for the design and development of biosensors, as shown in Figure 10. These factors can be satisfied by using nanotechnology‐enabled tools that have been developed over the past few years in areas other than the detection of SARS‐CoV‐2. Especially, smart sensors enabled by nanotechnology are cost‐effective and exhibit rapid diagnosis of the respiratory virus, including SARS and MERS infections. The knowledge generated from this can be transferred for the development of smart diagnostic tools for quick and specific diagnosis of coronavirus proteins, antibodies and nucleic acids. This section will discuss the existing sensors for detecting other respiratory viruses, which can be potentially used to diagnose coronaviruses.

### 4.1 Nanoparticles‐based paper biosensors

Fluorescent and colorimetric assays are used to identify and diagnose viral nucleic acids in point‐of‐care applications. This was observed in the study carried out by Teengram et al., who developed a paper‐based colorimetric assay to detect DNA linked with MERS‐CoV, *Mycobacterium tuberculosis* (MTB), and *Human papillomavirus* (HPV).^104^ The team employed pyrrolidinyl peptide nucleic acid (acpcPNA) with a positive charge which linked itself to the C‐terminal of lysine as a probe and silver nanoparticles. The presence or absence of the target DNA and differences in Ag nanoparticle dispersion causes observable color changes in the sensor. This paper‐based colorimetric DNA multiplex sensor showed high selectivity against any single‐base mismatch, two‐base mismatch, and any change in noncomplementary target DNA. The sensor was reported to offer a low detection limit of 1.53, 1.27, and 1.03 nM against MERS‐CoV, MTB, and HPV, respectively.

In another study, Crooks et al. designed a hybrid microfluidic device using a disposable paper electrode and a 3D printed plastic chip.^105^ This device could perform electrochemical detection of magnetic bead‐silver nanoparticles bio conjugates exhibiting a LOD of 12 pM. These biosensors can perform quantitative and qualitative detection of viruses and their biomarkers. For quick immunomagnetic detection of myeloperoxidase (MPO) (a biomarker indicating viral infection), an electrochemical lateral flow device has been developed.^106^ It used the antibody‐modified magnetic beads and antibodies tagged with horseradish peroxidase. The biological sample is added to magnetic beads and detection antibodies for 5 min, after which the complex is added to a nitrocellulose strip. The authors noted that biomarkers could be detected with a LOD of 0.18 ng ml^−1^ within 15 min. As noted from the studies mentioned above, such paper‐based assays can detect respiratory viruses. Therefore, these paper‐based biosensors can be potentially used to detect COVID‐19 viruses. More details about the paper‐based biosensors for detection of coronaviruses is covered in a recent review.^107^ As noted from the studies mentioned above, such paper‐based assays can detect respiratory viruses. Therefore, these paper‐based biosensors can be potentially used to detect COVID‐19 viruses.

### 4.2 Nanoparticles‐based electrochemical biosensors

In electrochemical biosensors, the substance used for making the electrode surface plays a key role because it determines the sensor's performance. For instance, the double‐layered capacitance determines the detection limit of the sensor, while the electron transfer rate influences its sensitivity and time lag before the results. The conventional materials used for the fabrication of biosensors include carbon,^105^, ^108^, ^109^ silicon, graphene. and fluorine‐doped tin oxide. Nanomaterials and nanocomposites are better preferred in biosensors' development because of their contribution in enhancing the surface area of the biosensors.^42^ This causes more bio receptors to link to the analyte, which will give the sensor a better detection range and improve its sensitivity. This was clearly realized in the study of Han et al. who used this technique to detect H1N1, H5N1, and H7N9 viruses^110^ using ZnO nanorods grown in polydimethylsiloxane solution. The ZnO nanorods gave an immobilization density of the antibodies owing to their high surface area. This biosensor showed a LOD of 1 pg/ml and could distinguish between the different viruses.

#### 4.2.1 Immunosensors

Electrochemical immunosensors can also be used to detect the COVID‐19 respiratory viruses. Studies have been conducted to evaluate other kinds of respiratory viruses using immunosensors including COVID‐19.^111^ Fu et al created a self‐sacrificial label that could detect the virus, H5N1.^112^ It could immobilize secondary antibody on magnetic nanoparticles—which would be further used in a sandwich immunoassay‐based sensor. The magnetic nanoparticles were electrochemically changed into electroactive Prussian blue by producing electrons from water, which released Fe^3+^ ions from the magnetic nanoparticles. In the next step, Fe^3+^ is reduced to Fe^2+^ at a lower voltage. This method creates a porous Prussian blue analogue that uses low reactant concentration compared to traditional methods. This process even shows a high sensitivity with LOD = 0.0022 hemagglutination units.

In a study by Zhou et al., an immunosensor was created to detect HIV‐1 p24 antigen.^113^ They employed a novel nanocomposite based on [Ru(bpy)_3_]^2+^‐SiO_2_ compound and gold nanoparticles. This was attached to an anti‐p24 antibody placed on graphene. This composite also holds a large amount of ruthenium electro‐chemiluminescent activity that enhances the sensor's sensitivity. The LOD was observed to be 1 pg/ml and the sensor showed high selectivity against diverse proteins. However, this device needs a long incubation time of 150 min and takes a comparatively longer time than the currently used PCR‐based methods.

**FIGURE 11:**
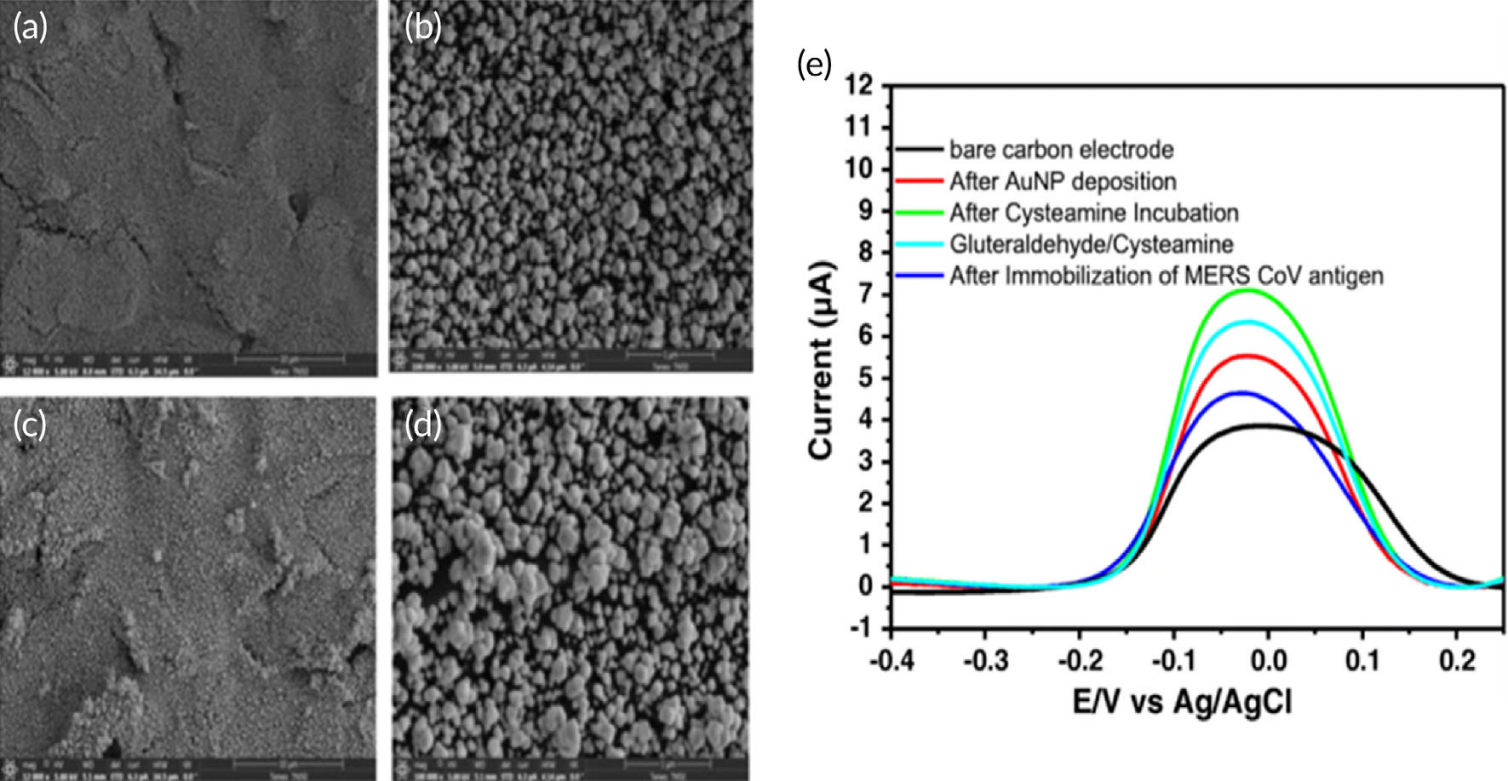
SEM images of Au nanoparticles (a, b) 20 CV scans and (c, d) 30 CV scans, and (e). Square wave voltammetry of MERS‐CoV immunosensor^114^

Layqah et al. developed an immunosensor that used the voltammetric detection method to diagnose MERS‐CoV.^114^ This biosensor uses disposable carbon microarray electrodes that are modified using gold nanoparticles. These nanoparticles increase the sensor's sensitivity and can be deposited easily on the carbon through electrochemical cycling. The surface of the electrode was modified with MERS‐CoV or HCoV antigens and a competition assay was used to detect the presence of the virus by addition of a foxed concentration of antibody that competes for the free virus and the antigen immobilized on the electrode surfaces. The sensor response was obtained by measuring the change in peak current using ferro/ferricyanide chemistry as a probe in square wave voltammetry (Figure 11). The detection limit was observed to be 0.4 for HCoV and 1.0 pg/ml for MERS‐CoV. The best aspect of this assay is that it takes under 20 min for detection.

Fluorescent immunosensors have also proven to be highly effective for the detection of several viruses. Weng et al. developed a nanostructured fluorescent immunosensor for the detection of infectious bronchitis virus (IBV) using a 2D nanosheet of molybdenum disulphide (MoS_2_).^115^ This biosensor was found to be inexpensive and could function based on FRET. The FRET was observed between the nanosystem and fluorescent dye when the Ab‐antigen immune complex was formed. As recorded by the authors, the sensitivity of the sensor was 4.6 × 10^2^ EID_50_/ml (median embryo infectious dose). At the same time, the detection limit was found to be between 10^2^ and 10^6^ EID_50_/ml.

## 5 NANOMATERIALS‐BASED SENSORS AND THEIR COMPARISON WITH THE CONVENTIONAL SENSORS

The recent nanobiotechnology developments have resulted in better methodologies and sensing techniques for diagnosing SARS‐CoV‐2 infection.^116^, ^117^, ^118^, ^119^ The unique properties of nanoparticles, like their high surface area‐to‐volume ratio, increased reactivity, and high adsorption, are essential for creating efficient biosensing techniques. Furthermore, the diameter and morphology of nanoparticles can be efficiently modified through covalent or noncovalent interactions to give distinct sensing properties, including low detection limit, increased sensitivity, selectivity, and rapid response.^93^ These attributes make nanomaterials‐based biosensors significantly different from conventional sensors.

One of the significant challenges for designing a highly sensitive and selective biosensor is to confer them to capture signals even with minute amounts. For this, the sensor must be susceptible to meager quantities of the target analytes. Nanomaterials are capable of amplifying signals at concentrations that are significantly low but are high enough to be detected and recorded owing to a large number of active sites on the surface of nanomaterials.^42^ Additionally, many metal nanoparticles like AuNPs, AgNPs or quantum dots like Cd, Pb label the target analyte by attaching to them.^65^, ^120^ With nano‐labeling, any electrochemical biosensor transforms into a highly selective labeled biosensor (Table 3).^65^

**TABLE 3:** Recently developed biosensors for the detection of coronavirus

Sensor electrode/sensor material selection	Concentration range of the sample	Detection limit	Time taken (sec)	Ref.
PMMA/graphene	‐	1.6 × 10^1^ pfu/ml	‐	10
FTO/AuNP	1 fM to 1 μM	90 fM	10–30	94
AuNI	0.01 pM to 50 μM	0.22 pM	‐	92
ASO/AuNPs	0.2 to 3 ng/μl	0.18 ng/μl	900	96
Au‐SPE/PDSM	10 fg to 1 μg/ml	1 fg/ml	180	121
mRT‐LAMP‐LFB		12 copies/reaction	3600	72
ssDNA‐conjugated AuNPs	585.4 copies/μl to 5.854 × 10^7^ copies/μl	6.9 copies/μl	300	122
PMMA	0.100 pg/ml to 1.00 ng/ml	1.00 pg/ml	‐	123
Quantum dots‐conjugated RNA aptamer	0.1 to 50 pg/ml	0.100 pg/ml	‐	124
Si_3_N_4_/SiO_2_	10^2^ to 10^3^ viruses/ml	‐	1800	125
SiNPs/Si‐PAA‐PSS	1 to 1 × 10^9^ copies/μl	1 copy/μl	3600	15
MNPs	0.1 to 10 fM	0.4 fM	5400	126
AuNPs	0.01 to 10,000 ng/ml	0.4 pg/ml	1200	114
Au‐TiO_2_‐Au	0 to 10^7^ vp/ml	370 vp/ml	900	127
Rutile prism/BK7/ITO film/tellurene/MoS_2_–COOH	~0 to 301.67 nM	8.4069 × 10^4^ deg/RIU	‐	128
AuNPs	‐	4.2 fmol	‐	81
AuNPs	93.3 pg/ml to 25.7 ng/ml	26.7 ± 7.7 pg/ml	1800	129
AuNPs	‐	0.08 ng/ml	1800	130
Graphene		1 fg/ml		131
Graphene/Cu	‐	0.2 pM	‐	132
Graphene	20 to 40 μg/ml (S1‐IgG)		600	133
TiO_2_	14 to 1400 nM	0.7 nM	30	134
CP/Au@Fe_3_O_4_		200 copies/ml		21
In_2_O_3_ nanowire		44 μM	600	135

*Note*: AuNI, two‐dimensional gold nanoislands; AuNP, gold nanoparticle; ASO, antisense oligonucleotides; FTO, fluorine‐doped tin oxide electrode; LFB, lateral flow biosensor; MNPs, magnetic nanoparticles; PDMS, polydimethylsiloxane; PMMA, Poly (methyl methacrylate); SPE, screen printed electrodes; vp, virus particles.

Thus, in comparison to conventional biosensors, nanoparticle‐based biosensors present various advantages as noted in Table 4. Conventional biosensors have been used to detect viral infections through the virus nucleic acid, antibody, aptamer, or antigen dependence.^136^ However, they lack the unique physicochemical properties of nanomaterials like their magnetic, optical, electrical, and optomagnetic properties. Traditional biosensors often require extensive specialized instruments for their fabrication and application, posing a challenge to its portability. Moreover, technologies involved in conventional biosensors require further steps for the samples to be processed at several levels before being analyzed. This thereby makes the whole process a little cumbersome and slower as compared to detection using nanomaterials‐based biosensors.^42^

**TABLE 4:** Different core nanoparticles used to develop biosensors based on different principles for SARS‐CoV‐2 virus detection

Core NPs	Biomarker	Principle
Gold NPs	Thiol‐c‐DNA receptor/nucleic acid	Plasmonic photothermal and localized surface plasmon resonance mediated biosensing
	Thiol‐mediated antisense oligonucleotides specific N‐gene of SARS‐CoV‐2	Plasmonic effect‐based colorimetric biosensing
	Oligo probe	Plasmonic effect‐based colorimetric biosensing
	Recombinant antigen of SARS‐CoV‐2 and rabbit‐IgG	lateral flow immunoassay‐based biosensing—colorimetric dependent
	nCOVID‐19 monoclonal antibody	Amperometric biosensing
Lanthanide doped polystyrene NPs	Anti‐human IgG antibody of mouse	Fluorescence biosensing—Lateral flow immunoassay
Graphene	Spike protein antibody	Amperometric biosensing—FET
Iron oxide NPs	Biotinylated probe	Optomagnetic biosensing
Polymer NPs coated with streptavidin	Rabbit anti‐fluorescein antibody, sheep anti‐digoxigenin antibody, and biotinylated BSA	mRT‐LAMP integrated with a nanoparticles‐based lateral flow biosensor assay

The impressive reduction in the size of the electrodes since the advent of nanotechnology has not only improved the biosensing properties but has also significantly increased their clinical applicability. In contrast to conventional technologies, nanoparticle‐based biosensors are time efficient and inexpensive. However, further work can be done to create nanomaterial‐based sensors such that analytes like a complementary single‐stranded nucleic acid aptamer can be linked. Aptamer‐based bifunctional biosensors are highly specific in targeting viral surface spike proteins S1, influencing the enzymatic processes to give electrochemical signals.^137^, ^138^

## 6 OTHER LATEST DISCOVERIES IN THE AREA OF COVID‐19 DIAGNOSTICS

### 6.1 CRISPR‐gene editing tool

Recently, many devices have been developed for rapid SARS‐CoV‐2 diagnostics. For instance, the Abbott COVID‐19 test was recently introduced and it gives results within 15 min and costs 1/20th of other test kits.^139^ Another California‐based team led by Dr Jennifer Doudna developed a test kit that can detect the SARS‐CoV‐2 in 5 min.^140^ This device uses a CRISPR‐gene editing technology that is linked to the cell phone camera. This is a revolutionary breakthrough in COVID‐19 diagnostics. This device could potentially reduce the period of COVID‐19 detection from several days to few minutes, thus making coronavirus testing possible even at homes. One of the biggest challenges this device faces is its reliability. However, as we discussed earlier, even techniques like PCR are prone to giving inaccurate results. Studies reveal that almost 30% of PCR tests may give false results.^141^ This new device uses portable additions like cheap laser illumination and collection optics and hence avoids the requirement of enormous laboratory apparatus used by conventional methods for COVID‐19 diagnostics. The presence of many CRISPR strands present in the device enhances its sensitivity to evaluate not just the status of the sample. Still, it can also reveal the amount of viral matter present in the sample.

### 6.2 Breath test

A research company, Imec, revealed that it developed a novel coronavirus testing kit that can detect SARS‐CoV‐2 particles in a patient's exhaled breath in less than 5 minutes. Therefore, this testing will prove to be a more superior, faster, and entirely noninvasive diagnostic measure that will help detect the coronavirus in large‐scale populations. The device comprises a sample collector that collects the aerosol containing the virus particles and an analytical unit. The sample collector has also been said to allow for RT qPCR function. Very recently, The National University of Singapore researchers have developed another breath test to give rapid coronavirus test results in under a minute with 90% accuracy. The device contains high precision breath sampler that can detect volatile organic compounds in the patient's exhaled breath. The breath is then passed into a mass spectrometer that is attached to a machine learning software. The software then gives an overview of the VOC profile of the breath, followed by the final results. The entire process takes less than a minute. Because of its ability to give real‐time results, this device shows promising advantages over other existing coronavirus testing options.

### 6.3 Internet of medical things integrated biosensors

The SARS‐CoV‐2 pandemic has proven that this infection creates several complications in the body and affects the function of many organs. Though a lot of effort has been put recently toward developing rapid COVID testing kits, studies are still under process to better understand the possible therapeutic options to treat the condition.^142^ To combat any challenges posed during sensing and therapies, artificial intelligence (AI) and internet of medical things integrated sensors (IoMT) can prove to be very beneficial to detect SARS‐CoV‐2 and to study the individual data and compare with a more extensive profile for a better and intelligent healthcare management system.^131^, ^143^ AI has proven helpful in analysis of medical imaging modalities such as ultrasound, X‐ray, and computed tomography. The rapid analysis powered by AI has helped in early diagnosis of the disease and has been reviewed in detail.^25^ Even though IoMTs are not directly capable of detecting an infection, the continuous monitoring of individual health data facilitates the detection of unfamiliar flare‐ups in body temperature, heart rate, and breathing patterns that are not possible to diagnose in infrequent doctor visits. The changes in the routine body patterns could be useful in alerting the individuals to perform a covid test. This may help in detecting asymptomatic yet contagious individuals and prevent the spread of the disease. In addition, IoMT will help establish better sensing alternatives that could allow wireless communication similar to “telemedicine.” For instance, in a recent study by Rodriguez et al., an IoT‐based biosensor was developed to detect SARS‐CoV‐2 biomarkers rapidly.^133^ This device, “RapidPlex,” is an electrochemical platform that can detect and monitor four SARS‐CoV‐2 antigens, including IgM, IgG, inflammatory C‐reactive protein, and the nucleocapsid protein in a given sample using laser engraved graphene electrodes. The integrated device could relay the test results to a smart phone and can be shared with health professionals that allows better management of the disease and prevent its further spread. Such integrated devices hold promise for the development of large platforms that can help in personalized pandemic management.

## 7 CONCLUSIONS AND FUTURE PERSPECTIVE

The asymptomatic cases and the complex epidemiology of SARS‐CoV‐2 have prompted researchers worldwide to create quick, selective, sensitive, inexpensive, portable, and highly reliable sensors for coronavirus diagnosis.^144^ The primary consideration is identifying the analyte without involving signal‐reporting agents, extraction, or incubation steps. Most of the biosensors developed for COVID‐19 detection require highly specific surface nucleoproteins attached to host angiotensin‐converting enzyme 2 (ACE‐2) receptors.^67^ Several such biomarkers from human hosts are used to develop effective biosensors. These include hematological biomarkers like lymphocyte count and neutrophil count, inflammatory biomarkers like procalcitonin or C‐reactive protein, immunological biomarkers like interleukin‐6 biochemical biomarkers like creatine kinase, aspartate aminotransferase (AST), among others.

As discussed in this review, sensors are a viable option for SARS‐COV‐2 detection. The conventional methods of detection are time‐consuming and are not very sensitive, thus may give false‐positive results. Furthermore, challenges like the rapid mutation rate of coronavirus and mass population testing, demand a quick and accurate SARS‐CoV‐2 diagnosis. Nanomaterial‐based biosensors show a considerable advantage in such a scenario. Sensors employing elements like gold‐ and carbon‐based nanomaterials have proven to be far more efficient than conventional sensors, as discussed in this review. Electrochemical biosensors are also popular in COVID‐19 detection based on the type of antibodies, aptamers, and imprinted polymers.^116^ Biosensors based on aptamers have also shown high sensitivity toward viral particles, like the spike protein S1 and have led to the regulation of the enzymatic action in electrochemical sensors.^137^ However, contamination of bioreceptors is a critical factor that hinders the sensor sensitivity, thereby yielding false results. Characteristic features of antigen, protein, antibody, nanomaterial type, and other factors can also affect the sensitivity and selectivity of the biosensor.

Hence, alternatives like CRISPR can be coupled with nanomaterial‐based biosensors to increase their selectivity in all kinds of samples like urine, blood, sputum, and nasopharyngeal swab.^145^ However, a plethora of false positive results from the improper nasopharyngeal swab testing calls for an effective and reliable sampling of the swabs. For starters, only synthetic fiber swabs with a flexible shaft must be used for specimen collection. The nasopharyngeal swab must be taken with utmost precaution. The non‐toxic synthetic fiber swab must be put deep inside the nasal cavity to obtain the clean samples. Then necessary safety precautions must be taken to process the samples accurately during the testing.^146^ Nanostructures like plasmonics have shown promising potential to be used in electrochemical biosensors to produce reliable and reproducible results during such assessments for COVID‐19 detection.^61^, ^80^ Such nanomaterials and nucleic acid molecules could be developed to detect viral particles in biological samples. Nonlabeling methodologies like SPR, Surface‐Enhanced Raman Scattering (SERS), and Quartz‐Crystal Microbalance (QCM) employing nanoparticles are promising techniques for creating biosensors for COVID‐19 detection.^147^ The Ag‐NPs hybridization techniques in quartz crystal microbalance DNA‐QCM sensing system can also be used to spot the coronavirus.^148^ Besides, techniques like localized surface plasmon‐coupled fluorescence (LSPCF) fiber optic biosensors can be used to detect SARS‐CoV‐2.^123^ Unique thiolated DNA capture probe sequences can be immobilized on screen‐printed electrodes surfaces and further linked to biotinylated target strand DNA. This was previously seen in the work of Ilkhani and the team for Ebola virus detection,^149^ and a similar strategy can be applied for COVID‐19 detection.

Thus it is clear that nanomaterial‐based biosensors offer several advantages for the detection of viral infections as opposed to the conventional testing methods. As discussed in this review, biosensors make the detection process more effective by increasing the sensitivity and selectivity of the sensors, reducing the response time, and can be easily miniaturized in the form of a portable point of care devices.^150^ Despite these unique advantages, nanomaterial‐based sensors for COVID‐19 detection have to address a few shortcomings before being available and acceptable at a commercial scale. More work needs to be done to increase the accuracy of the SARS‐CoV‐2 detection and reduce the rate of false positives. Similarly, unique advantages of the POC sensors enabled by nanotechnology should be supplemented with innovative options like integrating with artificial intelligence and the Internet of Things can also increase the predictability of false‐positive results and enhance the reproducibility of results.^142^, ^151^ In addition, machine learning‐based programs can aid in rapid signal processing during the detection process and help in obtaining direct and more accurate results. It is also important to increase the basic understanding of the interaction of nanomaterials with various functionalities to enhance their detection abilities using different spectroscopy and electronic tools. A combination of the knowledge derived from both theory and experiments will be required for pushing the frontier of advanced nanomaterials‐based biosensors for COVID‐19 detection.

## AUTHOR CONTRIBUTIONS

**Gowhar A. Naikoo:** Conceptualization (lead); writing – original draft (lead). **Fareeha Arshed:** Writing – original draft (supporting). **Israr U. Hassan:** Writing – original draft (supporting). **Tasbiha Awan:** Writing – original draft (supporting). **Hiba Salim:** Writing – original draft (supporting). **Mona Z. Pedram:** Writing – original draft (supporting). **Waqar Ahmed:** Writing – original draft (supporting). **Vaishwik Patel:** Visualization (lead); writing – original draft (supporting). **Ajay S. Karakoti:** Writing – review and editing (equal). **Ajayan Vinu:** Writing – review and editing (lead).

## CONFLICT OF INTEREST

There are no conflicts of interest to declare.

## Data Availability

Data sharing not applicable to this article as no datasets were generated or analysed during the current study being a review.

## References

[1] Zhou P , Yang X‐L , Wang X‐G , et al. A pneumonia outbreak associated with a new coronavirus of probable bat origin. Nature. 2020;579(7798):270‐273. 32015507 10.1038/s41586-020-2012-7 PMC7095418

[2] Lu R , Zhao X , Li J , et al. Genomic characterisation and epidemiology of 2019 novel coronavirus: implications for virus origins and receptor binding. Lancet. 2020;395(10224):565‐574. 32007145 10.1016/S0140-6736(20)30251-8 PMC7159086

[3] Chan JF‐W , Yuan S , Kok K‐H , et al. A familial cluster of pneumonia associated with the 2019 novel coronavirus indicating person‐to‐person transmission: a study of a family cluster. Lancet. 2020;395(10223):514‐523. 31986261 10.1016/S0140-6736(20)30154-9 PMC7159286

[4] Bohk‐Ewald C , Dudel C , Myrskylä M . A demographic scaling model for estimating the total number of COVID‐19 infections. Int J Epidemiol. 2020;49(6):1963‐1971. 10.1093/ije/dyaa198 PMC7799106 33349859

[5] Nicola M , Alsafi Z , Sohrabi C , et al. The socio‐economic implications of the coronavirus pandemic (COVID‐19): a review. Int J Surg. 2020;78:185‐193. 32305533 10.1016/j.ijsu.2020.04.018 PMC7162753

[6] Kumar N , Shetti NP , Jagannath S , Aminabhavi TM . Electrochemical sensors for the detection of SARS‐CoV‐2 virus. Chem Eng J. 2022;430:132966. 34690533 10.1016/j.cej.2021.132966 PMC8525496

[7] Xu L , Li D , Ramadan S , Li Y , Klein N . Facile biosensors for rapid detection of COVID‐19. Biosens Bioelectron. 2020;170:112673. 33038584 10.1016/j.bios.2020.112673 PMC7528898

[8] Pishva P , Yüce M . Nanomaterials to tackle the COVID‐19 pandemic. Emergent Mater. 2021;4(1):211‐229. 33615139 10.1007/s42247-021-00184-8 PMC7880038

[9] Zhou Z , Zhang Y , Shen Y , Liu S , Zhang Y . Molecular engineering of polymeric carbon nitride: advancing applications from photocatalysis to biosensing and more. Chem Soc Rev. 2018;47(7):2298‐2321. 29517786 10.1039/c7cs00840f

[10] Seo G , Lee G , Kim MJ , et al. Rapid detection of COVID‐19 causative virus (SARS‐CoV‐2) in human nasopharyngeal swab specimens using field‐effect transistor‐based biosensor. ACS Nano. 2020;14(4):5135‐5142. 32293168 10.1021/acsnano.0c02823

[11] Wang Z , Wei W , Shen Y , Liu S , Zhang Y . Carbon nitride–based biosensors. In: Biochemical sensors: nanomaterial‐based biosensing and application in honor of the 90th birthday of Prof. Shaojun Dong. World Scientific; 2021:175‐225.

[12] Shan B , Broza YY , Li W , et al. Multiplexed nanomaterial‐based sensor array for detection of COVID‐19 in exhaled breath. ACS Nano. 2020;14(9):12125‐12132. 32808759 10.1021/acsnano.0c05657

[13] Health, N. I. o. NIH‐wide strategic plan for COVID‐19 research. 2020.

[14] Sharma PK , Kim E‐S , Mishra S , et al. Ultrasensitive and reusable graphene oxide‐modified double‐interdigitated capacitive (DIDC) sensing chip for detecting SARS‐CoV‐2. ACS Sens. 2021;6(9):3468‐3476. 34478270 10.1021/acssensors.1c01437

[15] Chaibun T , Puenpa J , Ngamdee T , et al. Rapid electrochemical detection of coronavirus SARS‐CoV‐2. Nat Commun. 2021;12(1):1‐10. 33547323 10.1038/s41467-021-21121-7 PMC7864991

[16] Zhao Y , Chen J , Hu Z , et al. All‐solid‐state SARS‐CoV‐2 protein biosensor employing colloidal quantum dots‐modified electrode. Biosens Bioelectron. 2022;202:113974. 35032920 10.1016/j.bios.2022.113974 PMC8741628

[17] Gao J , Wang C , Wang C , et al. Poly‐l‐lysine‐modified graphene field‐effect transistor biosensors for ultrasensitive breast cancer miRNAs and SARS‐CoV‐2 RNA detection. Anal Chem. 2022;94:1626‐1636. 35025203 10.1021/acs.analchem.1c03786

[18] Heo W , Lee K , Park S , Hyun K‐A , Jung H‐I . Electrochemical biosensor for nucleic acid amplification‐free and sensitive detection of severe acute respiratory syndrome coronavirus 2 (SARS‐CoV‐2) RNA via CRISPR/Cas13a trans‐cleavage reaction. Biosens Bioelectron. 2022;201:113960. 35016109 10.1016/j.bios.2021.113960 PMC8730380

[19] Grabovenko F , Nikiforova L , Yanenko B , et al. Glycosylation of receptor binding domain of SARS‐CoV‐2 S‐protein influences on binding to immobilized DNA Aptamers. Int J Mol Sci. 2022;23, (1):557. 35008982 10.3390/ijms23010557 PMC8745424

[20] Wang S‐H , Kuo C‐W , Lo S‐C , Yeung WK , Chang T‐W , Wei P‐K . Spectral image contrast‐based flow digital nanoplasmon‐metry for ultrasensitive antibody detection. J Nanobiotechnol. 2022;20(1):1‐13. 10.1186/s12951-021-01188-6 PMC8724237 34983543

[21] Zhao H , Liu F , Xie W , et al. Ultrasensitive supersandwich‐type electrochemical sensor for SARS‐CoV‐2 from the infected COVID‐19 patients using a smartphone. Sensors Actuators B Chem. 2021;327:128899. 10.1016/j.snb.2020.128899 PMC7489230 32952300

[22] Roberts A , Chouhan RS , Shahdeo D , Shrikrishna NS , Kesarwani V , Horvat M , Gandhi S . A Recent Update on Advanced Molecular Diagnostic Techniques for COVID‐19 Pandemic: An Overview. Front Immunol. 2021;12:5316–5331. 10.3389/fimmu.2021.732756 PMC8712736 34970254

[23] Alathari, M. J. A. ; Al Mashhadany, Y. ; Mokhtar, M. H. H. ; Burham, N. ; Bin Zan, M. S. D. ; A Bakar , A. A.; Arsad, N. , Human body performance with COVID‐19 affectation according to virus specification based on biosensor techniques. Sensors 2021, 21, (24), 8362. 34960456 10.3390/s21248362 PMC8704003

[24] Ribeiro BV , Cordeiro TAR , e Freitas GRO , Ferreira LF , Franco DL . Biosensors for the detection of respiratory viruses: a review. Talanta Open. 2020;2:100007. 34913046 10.1016/j.talo.2020.100007 PMC7428963

[25] Gudigar A , Raghavendra U , Nayak S , et al. Role of artificial intelligence in COVID‐19 detection. Sensors. 2021;21(23):8045. 34884045 10.3390/s21238045 PMC8659534

[26] Szunerits S , Pagneux Q , Swaidan A , et al. The role of the surface ligand on the performance of electrochemical SARS‐CoV‐2 antigen biosensors. Anal Bioanal Chem. 2021;414:1‐11. 10.1007/s00216-020-03137-y PMC7897554 33616686

[27] Bisht A , Mishra A , Bisht H , Tripathi R . Nanomaterial based biosensors for detection of viruses including SARS‐CoV‐2: a review. J Anal Test. 2021;5(4):327‐340. 34777896 10.1007/s41664-021-00200-0 PMC8572656

[28] Verma MK , Sharma PK , Verma HK , et al. Rapid diagnostic methods for SARS‐CoV‐2 (COVID‐19) detection: an evidence‐based report. J Med Life. 2021;14, (4):431. 34621365 10.25122/jml-2021-0168 PMC8485368

[29] Samson R , Navale GR , Dharne MS . Biosensors: frontiers in rapid detection of COVID‐19. 3 Biotech. 2020;10(9):1‐9. 32818132 10.1007/s13205-020-02369-0 PMC7417775

[30] Parihar A , Ranjan P , Sanghi SK , Srivastava AK , Khan R . Point‐of‐care biosensor‐based diagnosis of COVID‐19 holds promise to combat current and future pandemics. ACS Appl Bio Mater. 2020;3(11):7326‐7343. 10.1021/acsabm.0c01083 35019474

[31] Alpdagtas S , Ilhan E , Uysal E , Sengor M , Ustundag CB , Gunduz O . Evaluation of current diagnostic methods for COVID‐19. APL Bioeng. 2020;4, (4):041506. 33305162 10.1063/5.0021554 PMC7710383

[32] Kopp MU , Mello AJ , Manz A . Chemical amplification: continuous‐flow PCR on a chip. Science. 1998;280(5366):1046‐1048. 9582111 10.1126/science.280.5366.1046

[33] Hashimoto M , Barany F , Soper SA . Polymerase chain reaction/ligase detection reaction/hybridization assays using flow‐through microfluidic devices for the detection of low‐abundant DNA point mutations. Biosens Bioelectron. 2006;21(10):1915‐1923. 16488597 10.1016/j.bios.2006.01.014

[34] Krishnan M , Ugaz VM , Burns MA . PCR in a Rayleigh‐Benard convection cell.(biochemistry). Science. 2002;298(5594):793‐794. 12399582 10.1126/science.298.5594.793

[35] Schneegaß I , Bräutigam R , Köhler JM . Miniaturized flow‐through PCR with different template types in a silicon chip thermocycler. Lab Chip. 2001;1(1):42‐49. 15100888 10.1039/b103846j

[36] Prakash AR , Adamia S , Sieben V , Pilarski P , Pilarski L , Backhouse C . Small volume PCR in PDMS biochips with integrated fluid control and vapour barrier. Sensors Actuators B Chem. 2006;113(1):398‐409.

[37] Christensen TB , Bang DD , Wolff A . Multiplex polymerase chain reaction (PCR) on a SU‐8 chip. Microelectron Eng. 2008;85(5–6):1278‐1281.

[38] Zhang C , Xu J , Ma W , Zheng W . PCR microfluidic devices for DNA amplification. Biotechnol Adv. 2006;24:243‐284. 16326063 10.1016/j.biotechadv.2005.10.002

[39] Liu H‐B , Gong H‐Q , Ramalingam N , Jiang Y , Dai C‐C , Hui KM . Micro air bubble formation and its control during polymerase chain reaction (PCR) in polydimethylsiloxane (PDMS) microreactors. J Micromech Microeng. 2007;17(10):2055‐2064.

[40] Trung NB , Saito M , Takabayashi H , Viet PH , Tamiya E , Takamura Y . Multi‐chamber PCR chip with simple liquid introduction utilizing the gas permeability of polydimethylsiloxane. Sensors Actuators B Chem. 2010;149(1):284‐290.

[41] Guliy O , Zaitsev B , Larionova O , Borodina I . Virus detection methods and biosensor technologies. Biophysics. 2019;64(6):890‐897.

[42] Naikoo GA , Awan T , Hassan IU , et al. Nanomaterials based sensors for respiratory viral detection: a review. IEEE Sensors J. 2021;21:17643‐17656. 10.1109/JSEN.2021.3085084 PMC8769020 35790098

[43] Wang YC , Lee YT , Yang T , Sun JR , Shen CF , Cheng CM . Current diagnostic tools for coronaviruses–from laboratory diagnosis to POC diagnosis for COVID‐19. Bioeng Transl Med. 2020;5(3):e10177. 32838038 10.1002/btm2.10177 PMC7435577

[44] Mujawar MA , Gohel H , Bhardwaj SK , Srinivasan S , Hickman N , Kaushik A . Nano‐enabled biosensing systems for intelligent healthcare: towards COVID‐19 management. Mater Today Chem. 2020;17:100306. 32835155 10.1016/j.mtchem.2020.100306 PMC7274574

[45] Udugama B , Kadhiresan P , Kozlowski HN , et al. Diagnosing COVID‐19: the disease and tools for detection. ACS Nano. 2020;14(4):3822‐3835. 32223179 10.1021/acsnano.0c02624

[46] Corman VM , Landt O , Kaiser M , et al. Detection of 2019 novel coronavirus (2019‐nCoV) by real‐time RT‐PCR. Euro Surveill. 2020;25(3):2000045. 10.2807/1560-7917.ES.2020.25.3.2000045 PMC6988269 31992387

[47] Bruning A , Aatola H , Toivola H , et al. Rapid detection and monitoring of human coronavirus infections. New Microbes New Infect. 2018;24:52‐55. 29872531 10.1016/j.nmni.2018.04.007 PMC5986163

[48] Gaunt ER , Hardie A , Claas EC , Simmonds P , Templeton KE . Epidemiology and clinical presentations of the four human coronaviruses 229E, HKU1, NL63, and OC43 detected over 3 years using a novel multiplex real‐time PCR method. J Clin Microbiol. 2010;48(8):2940‐2947. 20554810 10.1128/JCM.00636-10 PMC2916580

[49] Yuan X , Yang C , He Q , et al. Current and perspective diagnostic techniques for COVID‐19. ACS Infect Dis. 2020;6(8):1998‐2016. 32677821 10.1021/acsinfecdis.0c00365

[50] Zhao J , Yuan Q , Wang H , et al. Antibody responses to SARS‐CoV‐2 in patients with novel coronavirus disease 2019. Clin Infect Dis. 2020;71(16):2027‐2034. 32221519 10.1093/cid/ciaa344 PMC7184337

[51] Basu A , Zinger T , Inglima K , et al. Performance of Abbott ID now COVID‐19 rapid nucleic acid amplification test using nasopharyngeal swabs transported in viral transport media and dry nasal swabs in a New York City academic institution. J Clin Microbiol. 2020;58(8):e01136‐e01120. 32471894 10.1128/JCM.01136-20 PMC7383552

[52] Zhen W , Smith E , Manji R , Schron D , Berry GJ . Clinical evaluation of three sample‐to‐answer platforms for detection of SARS‐CoV‐2. J Clin Microbiol. 2020;58(8):e00783‐e00720. 32332061 10.1128/JCM.00783-20 PMC7383520

[53] Control, C. f. D. ; Prevention, Interim guidelines for collecting, handling, and testing clinical specimens for COVID‐19. 2020.

[54] Sullivan CB , Schwalje AT , Jensen M , et al. Cerebrospinal fluid leak after nasal swab testing for coronavirus disease 2019. JAMA Otolaryngol Head Neck Surg. 2020;146(12):1179‐1181. 33022069 10.1001/jamaoto.2020.3579

[55] Gupta K , Bellino PM , Charness ME . Adverse effects of nasopharyngeal swabs: three‐dimensional printed versus commercial swabs. Infect Control Hosp Epidemiol. 2021;42(5):641‐642. 32522313 10.1017/ice.2020.297 PMC7308627

[56] Alcusky M , McManus DD , Hume AL , Fisher M , Tjia J , Lapane KL . Changes in anticoagulant utilization among United States nursing home residents with atrial fibrillation from 2011 to 2016. J Am Heart Assoc. 2019;8(9):e012023. 31046504 10.1161/JAHA.119.012023 PMC6512099

[57] To KK‐W , Tsang OT‐Y , Leung W‐S , et al. Temporal profiles of viral load in posterior oropharyngeal saliva samples and serum antibody responses during infection by SARS‐CoV‐2: an observational cohort study. Lancet Infect Dis. 2020;20(5):565‐574. 32213337 10.1016/S1473-3099(20)30196-1 PMC7158907

[58] Wang W , Xu Y , Gao R , et al. Detection of SARS‐CoV‐2 in different types of clinical specimens. JAMA. 2020;323(18):1843‐1844. 32159775 10.1001/jama.2020.3786 PMC7066521

[59] Arevalo‐Rodriguez I , Buitrago‐Garcia D , Simancas‐Racines D , et al. False‐negative results of initial RT‐PCR assays for COVID‐19: a systematic review. PLoS One. 2020;15(12):e0242958. 33301459 10.1371/journal.pone.0242958 PMC7728293

[60] Laghrib F , Saqrane S , El Bouabi Y , et al. Current progress on COVID‐19 related to biosensing technologies: new opportunity for detection and monitoring of viruses. Microchem J. 2021;160:105606. 33052148 10.1016/j.microc.2020.105606 PMC7543751

[61] Shrivastav AM , Cvelbar U , Abdulhalim I . A comprehensive review on plasmonic‐based biosensors used in viral diagnostics. Commun Biol. 2021;4(1):1‐12. 33452375 10.1038/s42003-020-01615-8 PMC7810758

[62] Yüce M , Filiztekin E , Özkaya KG . COVID‐19 diagnosis—a review of current methods. Biosens Bioelectron. 2021;172:112752. 33126180 10.1016/j.bios.2020.112752 PMC7584564

[63] Zhang N , Wang L , Deng X , et al. Recent advances in the detection of respiratory virus infection in humans. J Med Virol. 2020;92(4):408‐417. 31944312 10.1002/jmv.25674 PMC7166954

[64] Alizadeh N , Salimi A . Ultrasensitive bioaffinity electrochemical sensors: advances and new perspectives. Electroanalysis. 2018;30(12):2803‐2840.

[65] Saylan Y , Erdem Ö , Ünal S , Denizli A . An alternative medical diagnosis method: biosensors for virus detection. Biosensors. 2019;9(2):65. 10.3390/bios9020065 PMC6627152 31117262

[66] Narita F , Wang Z , Kurita H , et al. A review of piezoelectric and magnetostrictive biosensor materials for detection of COVID‐19 and other viruses. Adv Mater. 2021;33, (1):2005448. 10.1002/adma.202005448 PMC7744850 33230875

[67] Liu Z , Xiao X , Wei X , et al. Composition and divergence of coronavirus spike proteins and host ACE2 receptors predict potential intermediate hosts of SARS‐CoV‐2. J Med Virol. 2020;92(6):595‐601. 32100877 10.1002/jmv.25726 PMC7228221

[68] Chen L , Xiong J , Bao L , Shi Y . Convalescent plasma as a potential therapy for COVID‐19. Lancet Infect Dis. 2020;20(4):398‐400. 32113510 10.1016/S1473-3099(20)30141-9 PMC7128218

[69] Park G‐S , Ku K , Baek S‐H , et al. Development of reverse transcription loop‐mediated isothermal amplification assays targeting severe acute respiratory syndrome coronavirus 2 (SARS‐CoV‐2). J Mol Diagn. 2020;22(6):729‐735. 32276051 10.1016/j.jmoldx.2020.03.006 PMC7144851

[70] Yu L , Wu S , Hao X , et al. Rapid detection of COVID‐19 coronavirus using a reverse transcriptional loop‐mediated isothermal amplification (RT‐LAMP) diagnostic platform. Clin Chem. 2020;66(7):975‐977. 32315390 10.1093/clinchem/hvaa102 PMC7188121

[71] Kashir J , Yaqinuddin A . Loop mediated isothermal amplification (LAMP) assays as a rapid diagnostic for COVID‐19. Med Hypotheses. 2020;141:109786. 32361529 10.1016/j.mehy.2020.109786 PMC7182526

[72] Zhu X , Wang X , Han L , et al. Multiplex reverse transcription loop‐mediated isothermal amplification combined with nanoparticle‐based lateral flow biosensor for the diagnosis of COVID‐19. Biosens Bioelectron. 2020;166:112437. 32692666 10.1016/j.bios.2020.112437 PMC7361114

[73] Amaral C , Antunes W , Moe E , et al. A molecular test based on RT‐LAMP for rapid, sensitive and inexpensive colorimetric detection of SARS‐CoV‐2 in clinical samples. Sci Rep. 2021;11(1):1‐12. 34385527 10.1038/s41598-021-95799-6 PMC8361189

[74] Galvez LC , Barbosa CFC , Koh RBL , Aquino VM . Loop‐mediated isothermal amplification (LAMP) assays for the detection of abaca bunchy top virus and banana bunchy top virus in abaca. Crop Prot. 2020;131:105101.

[75] Zhang F , Abudayyeh OO , Gootenberg JS . A protocol for detection of COVID‐19 using CRISPR diagnostics. Vol 8; Synthego; 2020.

[76] Hajian R , Balderston S , Tran T , et al. Detection of unamplified target genes via CRISPR–Cas9 immobilized on a graphene field‐effect transistor. Nat Biomed Eng. 2019;3(6):427‐437. 31097816 10.1038/s41551-019-0371-x PMC6556128

[77] Broughton JP , Deng X , Yu G , et al. CRISPR–Cas12‐based detection of SARS‐CoV‐2. Nat Biotechnol. 2020;38(7):870‐874. 32300245 10.1038/s41587-020-0513-4 PMC9107629

[78] Lee T , Ahn J‐H , Park SY , et al. Recent advances in AIV biosensors composed of nanobio hybrid material. Micromachines (Basel). 2018;9(12):651. 10.3390/mi9120651 PMC6316213 30544883

[79] Djaileb A , Charron B , Jodaylami MH , et al. A Rapid and Quantitative Serum Test for SARS‐CoV‐2 Antibodies with Portable Surface Plasmon Resonance Sensing. ChemRxiv; 2020.

[80] Ahmadivand A , Gerislioglu B , Ramezani Z , Kaushik A , Manickam P , Ghoreishi SA . Functionalized terahertz plasmonic metasensors: Femtomolar‐level detection of SARS‐CoV‐2 spike proteins. Biosens Bioelectron. 2021;177:112971. 33434777 10.1016/j.bios.2021.112971 PMC7787065

[81] Ahmadivand A , Gerislioglu B , Ramezani Z , Kaushik A , Manickam P , Ghoreishi SA . Femtomolar‐level detection of SARS‐CoV‐2 spike proteins using toroidal plasmonic metasensors. arXiv Preprint arXiv:200608536. 2020. [Preprint] 10.1016/j.bios.2021.112971 PMC7787065 33434777

[82] Broza YY , Vishinkin R , Barash O , Nakhleh MK , Haick H . Synergy between nanomaterials and volatile organic compounds for non‐invasive medical evaluation. Chem Soc Rev. 2018;47(13):4781‐4859. 29888356 10.1039/c8cs00317c

[83] Ebralidze II , Laschuk NO , Poisson J , Zenkina OV . Colorimetric sensors and sensor arrays. Nanomaterials design for sensing applications. Elsevier; 2019:1‐39.

[84] Broza YY , Haick H . Nanomaterial‐based sensors for detection of disease by volatile organic compounds. Nanomedicine. 2013;8(5):785‐806. 23656265 10.2217/nnm.13.64

[85] Broza YY , Zhou X , Yuan M , et al. Disease detection with molecular biomarkers: from chemistry of body fluids to nature‐inspired chemical sensors. Chem Rev. 2019;119(22):11761‐11817. 31729868 10.1021/acs.chemrev.9b00437

[86] Adams E , Ainsworth M , Anand R , et al. Antibody testing for COVID‐19: A report from the National COVID Scientific Advisory Panel. Wellcome Open Res. 2020;5:139‐156. 33748431 10.12688/wellcomeopenres.15927.1 PMC7941096

[87] Whitman JD , Hiatt J , Mowery CT , et al. Test performance evaluation of SARS‐CoV‐2 serological assays. Nat Biotechnol. 2020;38(10):1174‐1183. 32855547 10.1038/s41587-020-0659-0 PMC7740072

[88] Chen Z , Zhang Z , Zhai X , et al. Rapid and sensitive detection of anti‐SARS‐CoV‐2 IgG, using lanthanide‐doped nanoparticles‐based lateral flow immunoassay. Anal Chem. 2020;92(10):7226‐7231. 32323974 10.1021/acs.analchem.0c00784

[89] Li Z , Yi Y , Luo X , et al. Development and clinical application of a rapid IgM‐IgG combined antibody test for SARS‐CoV‐2 infection diagnosis. J Med Virol. 2020;92(9):1518‐1524. 32104917 10.1002/jmv.25727 PMC7228300

[90] Ma Q , Wang J , Li Z , Lv X , Liang L , Yuan Q . Recent progress in time‐resolved biosensing and bioimaging based on lanthanide‐doped nanoparticles. Small. 2019;15(32):1804969. 10.1002/smll.201804969 30761729

[91] Banerjee R , Jaiswal A . Recent advances in nanoparticle‐based lateral flow immunoassay as a point‐of‐care diagnostic tool for infectious agents and diseases. Analyst. 2018;143(9):1970‐1996. 29645058 10.1039/c8an00307f

[92] Qiu G , Gai Z , Tao Y , Schmitt J , Kullak‐Ublick GA , Wang J . Dual‐functional plasmonic photothermal biosensors for highly accurate severe acute respiratory syndrome coronavirus 2 detection. ACS Nano. 2020;14(5):5268‐5277. 32281785 10.1021/acsnano.0c02439

[93] Maduraiveeran G , Sasidharan M , Ganesan V . Electrochemical sensor and biosensor platforms based on advanced nanomaterials for biological and biomedical applications. Biosens Bioelectron. 2018;103:113‐129. 29289816 10.1016/j.bios.2017.12.031

[94] Mahari S , Roberts A , Shahdeo D , Gandhi S . Ecovsens‐ultrasensitive novel in‐house built printed circuit board based electrochemical device for rapid detection of nCovid‐19 antigen, a spike protein domain 1 of SARS‐CoV‐2. bioRxiv. 2020. [Preprint]

[95] Draz MS , Shafiee H . Applications of gold nanoparticles in virus detection. Theranostics. 2018;8(7):1985‐2017. 29556369 10.7150/thno.23856 PMC5858513

[96] Moitra P , Alafeef M , Dighe K , Frieman MB , Pan D . Selective naked‐eye detection of SARS‐CoV‐2 mediated by N gene targeted antisense oligonucleotide capped plasmonic nanoparticles. ACS Nano. 2020;14(6):7617‐7627. 32437124 10.1021/acsnano.0c03822

[97] Barnett JM , Monnier BM , Tyler S , et al. Initial trail results of a magnetic biosensor for the rapid detection of porcine reproductive and respiratory virus (PRRSV) infection. Sens Bio‐Sens Res. 2020;27:100315.

[98] Islam MA , Ahsan MZ . Plausible approach for rapid detection of SARS‐CoV‐2 virus by magnetic nanoparticle based biosensors. Am J Nanosciences. 2020;6(2):6‐13.

[99] Perez JM , Simeone FJ , Saeki Y , Josephson L , Weissleder R . Viral‐induced self‐assembly of magnetic nanoparticles allows the detection of viral particles in biological media. J Am Chem Soc. 2003;125(34):10192‐10193. 12926940 10.1021/ja036409g

[100] Zheng X , Zhao L , Wen D , et al. Ultrasensitive fluorescent detection of HTLV‐II DNA based on magnetic nanoparticles and atom transfer radical polymerization signal amplification. Talanta. 2020;207:120290. 31594607 10.1016/j.talanta.2019.120290

[101] Tian B , Fock J , Minero GAS , Hansen MF . Nicking‐assisted on‐loop and off‐loop enzymatic cascade amplification for optomagnetic detection of a highly conserved dengue virus sequence. Biosens Bioelectron. 2020;160:112219. 32339155 10.1016/j.bios.2020.112219

[102] Khan M , Hasan M , Hossain S , Ahommed M , Daizy M . Ultrasensitive detection of pathogenic viruses with electrochemical biosensor: state of the art. Biosens Bioelectron. 2020;166:112431. 32862842 10.1016/j.bios.2020.112431 PMC7363606

[103] Zhao Z , Cui H , Song W , Ru X , Zhou W , Yu X . A simple magnetic nanoparticles‐based viral RNA extraction method for efficient detection of SARS‐CoV‐2. bioRxiv. 2020. [Preprint] 10.1016/j.talanta.2023.124479 PMC10035799 36966663

[104] Teengam P , Siangproh W , Tuantranont A , Vilaivan T , Chailapakul O , Henry CS . Multiplex paper‐based colorimetric DNA sensor using pyrrolidinyl peptide nucleic acid‐induced AgNPs aggregation for detecting MERS‐CoV, MTB, and HPV oligonucleotides. Anal Chem. 2017;89(10):5428‐5435. 28394582 10.1021/acs.analchem.7b00255 PMC7077925

[105] Walgama C , Nguyen MP , Boatner LM , Richards I , Crooks RM . Hybrid paper and 3D‐printed microfluidic device for electrochemical detection of Ag nanoparticle labels. Lab Chip. 2020;20(9):1648‐1657. 32255136 10.1039/d0lc00276c PMC7204514

[106] de Eguilaz MR , Cumba LR , Forster RJ . Electrochemical detection of viruses and antibodies: a mini review. Electrochem Commun. 2020;116:106762. 32501391 10.1016/j.elecom.2020.106762 PMC7247998

[107] Pinheiro T , Cardoso AR , Sousa CE , et al. Paper‐based biosensors for COVID‐19: a review of innovative tools for controlling the pandemic. ACS Omega. 2021;6(44):29268‐29290. 34778604 10.1021/acsomega.1c04012 PMC8577188

[108] Bandodkar AJ , Imani S , Nunez‐Flores R , et al. Re‐usable electrochemical glucose sensors integrated into a smartphone platform. Biosens Bioelectron. 2018;101:181‐187. 29073519 10.1016/j.bios.2017.10.019 PMC5841915

[109] Kaushik A , Yndart A , Kumar S , et al. A sensitive electrochemical immunosensor for label‐free detection of Zika‐virus protein. Sci Rep. 2018;8(1):1‐5. 29946074 10.1038/s41598-018-28035-3 PMC6018776

[110] Han J‐H , Lee D , Chew CHC , Kim T , Pak JJ . A multi‐virus detectable microfluidic electrochemical immunosensor for simultaneous detection of H1N1, H5N1, and H7N9 virus using ZnO nanorods for sensitivity enhancement. Sensors Actuators B Chem. 2016;228:36‐42.

[111] Roda A , Cavalera S , Di Nardo F , et al. Dual lateral flow optical/chemiluminescence immunosensors for the rapid detection of salivary and serum IgA in patients with COVID‐19 disease. Biosens Bioelectron. 2021;172:112765. 33126179 10.1016/j.bios.2020.112765 PMC7586100

[112] Zhang Q , Li L , Qiao Z , et al. Electrochemical conversion of Fe3O4 magnetic nanoparticles to electroactive Prussian blue analogues for self‐sacrificial label biosensing of avian influenza virus H5N1. Anal Chem. 2017;89(22):12145‐12151. 29053256 10.1021/acs.analchem.7b02784

[113] Zhou L , Huang J , Yu B , Liu Y , You T . A novel electrochemiluminescence immunosensor for the analysis of HIV‐1 p24 antigen based on P‐RGO@ Au@ Ru‐SiO2 composite. ACS Appl Mater Interfaces. 2015;7(44):24438‐24445. 26488492 10.1021/acsami.5b08154

[114] Layqah LA , Eissa S . An electrochemical immunosensor for the corona virus associated with the Middle East respiratory syndrome using an array of gold nanoparticle‐modified carbon electrodes. Microchim Acta. 2019;186(4):1‐10. 10.1007/s00604-019-3345-5 PMC7088225 30847572

[115] Weng X , Neethirajan S . Immunosensor based on antibody‐functionalized MoS 2 for rapid detection of avian coronavirus on cotton thread. IEEE Sensors J. 2018;18(11):4358‐4363. 10.1109/JSEN.2018.2829084 PMC7186039 32390783

[116] Cesewski E , Johnson BN . Electrochemical biosensors for pathogen detection. Biosens Bioelectron. 2020;159:112214. 32364936 10.1016/j.bios.2020.112214 PMC7152911

[117] Malhotra BD , Ali MA . Chapter 1 Nanomaterials in biosensors fundamentals and applications. Nanomaterials for biosensors. Elsevier; 2018.

[118] Palmieri V , Papi M . Can graphene take part in the fight against COVID‐19? Nano Today. 2020;33:100883. 32382315 10.1016/j.nantod.2020.100883 PMC7203038

[119] Ravina R , Dalal A , Mohan H , Prasad M , Pundir C . Detection methods for influenza a H1N1 virus with special reference to biosensors: a review. Biosci Rep. 2020;40:2, BSR20193852. 10.1042/BSR20193852 PMC7000365 32016385

[120] Arduini F , Micheli L , Moscone D , et al. Electrochemical biosensors based on nanomodified screen‐printed electrodes: recent applications in clinical analysis. TrAC Trends Anal Chem. 2016;79:114‐126.

[121] Mavrikou S , Moschopoulou G , Tsekouras V , Kintzios S . Development of a portable, ultra‐rapid and ultra‐sensitive cell‐based biosensor for the direct detection of the SARS‐CoV‐2 S1 spike protein antigen. Sensors. 2020;20(11):3121. 10.3390/s20113121 PMC7309076 32486477

[122] Alafeef M , Dighe K , Moitra P , Pan D . Rapid, ultrasensitive, and quantitative detection of SARS‐CoV‐2 using antisense oligonucleotides directed electrochemical biosensor chip. ACS Nano. 2020;14(12):17028‐17045. 10.1021/acsnano.0c06392 33079516

[123] Huang JC , Chang Y‐F , Chen K‐H , et al. Detection of severe acute respiratory syndrome (SARS) coronavirus nucleocapsid protein in human serum using a localized surface plasmon coupled fluorescence fiber‐optic biosensor. Biosens Bioelectron. 2009;25(2):320‐325. 19660929 10.1016/j.bios.2009.07.012 PMC7127111

[124] Roh C , Jo SK . Quantitative and sensitive detection of SARS coronavirus nucleocapsid protein using quantum dots‐conjugated RNA aptamer on chip. J Chem Technol Biotechnol. 2011;86(12):1475‐1479. 32336860 10.1002/jctb.2721 PMC7167159

[125] Ruiz‐Vega G , Soler M , Lechuga LM . Nanophotonic biosensors for point‐of‐care COVID‐19 diagnostics and coronavirus surveillance. J Phys Photonics. 2021;3(1):011002.

[126] Tian B , Gao F , Fock J , Dufva M , Hansen MF . Homogeneous circle‐to‐circle amplification for real‐time optomagnetic detection of SARS‐CoV‐2 RdRp coding sequence. Biosens Bioelectron. 2020;165:112356. 32510339 10.1016/j.bios.2020.112356

[127] Huang L , Ding L , Zhou J , et al. One‐step rapid quantification of SARS‐CoV‐2 virus particles via low‐cost nanoplasmonic sensors in generic microplate reader and point‐of‐care device. Biosens Bioelectron. 2021;171:112685. 33113383 10.1016/j.bios.2020.112685 PMC7557276

[128] Peng X , Zhou Y , Nie K , et al. Promising near‐infrared plasmonic biosensor employed for specific detection of SARS‐CoV‐2 and its spike glycoprotein. New J Phys. 2020;22(10):103046.

[129] Zhao B , Che C , Wang W , Li N , Cunningham BT . Single‐step, wash‐free digital immunoassay for rapid quantitative analysis of serological antibody against SARS‐CoV‐2 by photonic resonator absorption microscopy. Talanta. 2021;225:122004. 33592744 10.1016/j.talanta.2020.122004 PMC7833826

[130] Funari R , Chu K‐Y , Shen AQ . Detection of antibodies against SARS‐CoV‐2 spike protein by gold nanospikes in an opto‐microfluidic chip. Biosens Bioelectron. 2020;169:112578. 32911317 10.1016/j.bios.2020.112578 PMC7467868

[131] Kaushik AK , Dhau JS , Gohel H , et al. Electrochemical SARS‐CoV‐2 sensing at point‐of‐care and artificial intelligence for intelligent COVID‐19 management. ACS Appl Bio Mater. 2020;3(11):7306‐7325. 10.1021/acsabm.0c01004 35019473

[132] Gaurav A , Shukla P . Rapid detection of covid‐19 causative virus (sars‐cov‐2) using FET‐based biosensor. Int Res J Modernizat Eng Technol Sci. 2020;2:1207‐1214.

[133] Torrente‐Rodríguez RM , Lukas H , Tu J , et al. SARS‐CoV‐2 RapidPlex: a graphene‐based multiplexed telemedicine platform for rapid and low‐cost COVID‐19 diagnosis and monitoring. Matter. 2020;3(6):1981‐1998. 33043291 10.1016/j.matt.2020.09.027 PMC7535803

[134] Vadlamani BS , Uppal T , Verma SC , Misra M . Functionalized TiO2 nanotube‐based electrochemical biosensor for rapid detection of SARS‐CoV‐2. Sensors. 2020;20(20):5871. 10.3390/s20205871 PMC7589637 33080785

[135] Ishikawa FN , Chang H‐K , Curreli M , et al. Label‐free, electrical detection of the SARS virus N‐protein with nanowire biosensors utilizing antibody mimics as capture probes. ACS Nano. 2009;3(5):1219‐1224. 19422193 10.1021/nn900086c PMC2765574

[136] Ozer T , Geiss BJ , Henry CS . Chemical and biological sensors for viral detection. J Electrochem Soc. 2019;167(3):037523. 32287357 10.1149/2.0232003JES PMC7106559

[137] Acquah C , Danquah MK , Agyei D , Moy CK , Sidhu A , Ongkudon CM . Deploying aptameric sensing technology for rapid pandemic monitoring. Crit Rev Biotechnol. 2016;36(6):1010‐1022. 26381238 10.3109/07388551.2015.1083940

[138] Wang R , Xu L , Li Y . Bio‐nanogate controlled enzymatic reaction for virus sensing. Biosens Bioelectron. 2015;67:400‐407. 25212377 10.1016/j.bios.2014.08.071

[139] Bryan A , Pepper G , Wener MH , et al. Performance characteristics of the Abbott architect SARS‐CoV‐2 IgG assay and seroprevalence in Boise, Idaho. J Clin Microbiol. 2020;58(8):e00941‐e00920. 32381641 10.1128/JCM.00941-20 PMC7383515

[140] Fozouni P , Son S , Díaz de León Derby M , et al. Amplification‐free detection of SARS‐CoV‐2 with CRISPR‐Cas13a and mobile phone microscopy. Cell. 2021;184(2):323‐333.e9. 33306959 10.1016/j.cell.2020.12.001 PMC7834310

[141] Ramdas K , Darzi A , Jain S . ‘Test, re‐test, re‐test’: using inaccurate tests to greatly increase the accuracy of COVID‐19 testing. Nat Med. 2020;26(6):810‐811. 32398878 10.1038/s41591-020-0891-7 PMC7215136

[142] Jain S , Nehra M , Kumar R , et al. Internet of medical things (IoMT)‐integrated biosensors for point‐of‐care testing of infectious diseases. Biosens Bioelectron. 2021;179:113074. 33596516 10.1016/j.bios.2021.113074 PMC7866895

[143] Gadaleta M , Radin JM , Baca‐Motes K , et al. Passive detection of COVID‐19 with wearable sensors and explainable machine learning algorithms. NPJ Digit Med. 2021;4(1):1‐10. 34880366 10.1038/s41746-021-00533-1 PMC8655005

[144] Huffman JA , Perring AE , Savage NJ , et al. Real‐time sensing of bioaerosols: review and current perspectives. Aerosol Sci Technol. 2020;54(5):465‐495.

[145] Lucia C , Federico P‐B , Alejandra GC . An ultrasensitive, rapid, and portable coronavirus SARS‐CoV‐2 sequence detection method based on CRISPR‐Cas12. bioRxiv. 2020. [Preprint]

[146] Tang Y‐W , Schmitz JE , Persing DH , Stratton CW . Laboratory diagnosis of COVID‐19: current issues and challenges. J Clin Microbiol. 2020;58(6):e00512‐e00520. 32245835 10.1128/JCM.00512-20 PMC7269383

[147] Ngo HT , Wang H‐N , Fales AM , Vo‐Dinh T . Plasmonic SERS biosensing nanochips for DNA detection. Anal Bioanal Chem. 2016;408(7):1773‐1781. 26547189 10.1007/s00216-015-9121-4

[148] Chen S‐H , Chuang Y‐C , Lu Y‐C , Lin H‐C , Yang Y‐L , Lin C‐S . A method of layer‐by‐layer gold nanoparticle hybridization in a quartz crystal microbalance DNA sensing system used to detect dengue virus. Nanotechnology. 2009;20(21):215501. 19423930 10.1088/0957-4484/20/21/215501

[149] Ilkhani H , Farhad S . A novel electrochemical DNA biosensor for Ebola virus detection. Anal Biochem. 2018;557:151‐155. 29908157 10.1016/j.ab.2018.06.010

[150] Alhalaili B , Popescu IN , Kamoun O , Alzubi F , Alawadhia S , Vidu R . Nanobiosensors for the detection of novel coronavirus 2019‐nCoV and other pandemic/epidemic respiratory viruses: a review. Sensors. 2020;20, (22):6591. 10.3390/s20226591 PMC7698809 33218097

[151] Jeong H , Rogers JA , Xu S . Continuous on‐body sensing for the COVID‐19 pandemic: gaps and opportunities. Sci Adv. 2020;6, (36):eabd4794. 32917604 10.1126/sciadv.abd4794 PMC7467694

